# Design, Synthesis and Biochemical Evaluation of Novel Selective Estrogen Receptor Ligand Conjugates Incorporating an Endoxifen-Combretastatin Hybrid Scaffold

**DOI:** 10.3390/biomedicines4030015

**Published:** 2016-07-20

**Authors:** Niall O. Keely, Miriam Carr, Bassem Yassin, Gloria Ana, David G. Lloyd, Daniela Zisterer, Mary J. Meegan

**Affiliations:** 1 School of Pharmacy and Pharmaceutical Sciences, Trinity College Dublin, Dublin 2, Ireland; nkeely@tcd.ie (N.O.K.); carrmi@tcd.ie (M.C.); bassem76@yahoo.com (B.Y.); 2 School of Pharmacy and Pharmaceutical Sciences, Trinity Biomedical Sciences Institute, 152-160 Pearse Street, Trinity College Dublin, Dublin 2, Ireland; anag@tcd.ie; 3 School of Biochemistry and Immunology, Trinity Biomedical Sciences Institute, 152-160 Pearse Street, Trinity College Dublin, Dublin 2, Ireland; dzistrer@tcd.ie; 4 Division of Health Sciences, University of South Australia, Adelaide SA 5000, Australia; David.Lloyd@unisa.edu.au

**Keywords:** estrogen receptor ligands, selective estrogen receptor modulators, tumour targeting, conjugates, tamoxifen, endoxifen, hormone-dependent breast cancer

## Abstract

Nuclear-receptors are often overexpressed in tumours and can thereby be used as targets when designing novel selective chemotherapeutic agents. To date, many conjugates incorporating an estrogen receptor (ER) ligand have been synthesised in order to direct chemical agents to tissue sites containing ERs. A series of ER ligand conjugates were synthesised incorporating an antagonistic ER ligand scaffold based on endoxifen, covalently-bound via an amide linkage to a variety of combretastatin-based analogues, which may act as antimitotic agents. These novel endoxifen-combretastatin hybrid scaffold analogues were biochemically evaluated in order to determine their antiproliferative and cytotoxicity effects in both the ER-positive MCF-7 and the ER-negative MDA-MB-231 human breast cancer cell lines. ER competitive binding assays were carried out to assess the binding affinity of the lead conjugate **28** towards both the ERα and ERβ isoforms. In results from the NCI 60-cell line screen, the lead conjugate **28** displayed potent and highly selective antiproliferative activity towards the MCF-7 human cancer cell line (IC_50_ = 5 nM). In the ER-binding assays, the lead conjugate **28** demonstrated potent ER competitive binding in ERα (IC_50_ value: 0.9 nM) and ERβ (IC_50_ value: 4.7 nM). Preliminary biochemical results also demonstrate that the lead conjugate **28** may exhibit pure antagonism. This series makes an important addition to the class of ER antagonists and may have potential applications in anticancer therapy.

## 1. Introduction

**Figure 1:**
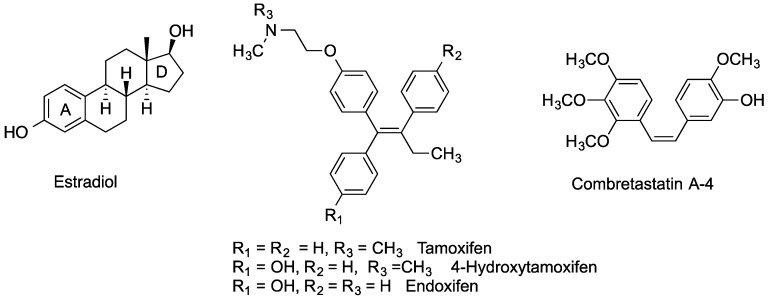
Estradiol, Tamoxifen, 4-Hydroxytamoxifen, Endoxifen and Combretastatin A-4.

Estrogen receptors (ER), principally present as two main isoforms; ERα and ERβ, are found in abundance in female reproductive tissues such as the breast, uterus and ovary, while also found in bone, liver and brain tissue [1,2,3,4,5]. ERs can be overexpressed in tumour tissue and this provides a means to selectively target these tissues by both steroidal and non-steroidal ER ligands. ER ligands can be classified by their agonistic and antagonistic behaviour in the different ER-isoforms [3,4,5,6]. The term selective estrogen receptor subtype modulator (SERSM) refers to the observation that a large number of reported ER-ligands have varying degrees of agonist/antagonistic behaviour towards the ERα and ERβ isoforms at the different tissue sites [3,4,5,6,7]. This leads to a complex action [1] where the benefits of a ligand at one ER-tissue site may be lessened by the negative effects the same ligand exerts at another ER-tissue site. For many decades, tamoxifen has been prescribed worldwide for the treatment of hormone-dependent breast cancer, (Figure 1). Tamoxifen displays antagonistic behaviour in breast tumour tissue; however this drug also displays agonistic behaviour on uterine tissue, which can lead to an increased risk of developing uterine cancer in postmenopausal women [3,4,5]. The other main concern in the use of tamoxifen is increased incidence of blood clots [8]. Breast cancer is often hormone dependent in its early stages of development. However as the disease progresses, the tumours can become less hormone dependent and difficult to treat [9,10]. For an effective treatment of hormone-dependent breast cancer, one goal would be to design an ER-ligand with no noticeable agonistic effects, thus displaying pure antagonistic properties.

Various strategies attempt to improve the selectivity of chemotherapeutic agents by specifically targeting cancer cells and tumour environments [11,12]. Conjugates have been designed containing multiple pharmacophore elements or ligands, individually separated by a linker group, which aim to exert a synergistic and improved selective action on the target disease [13]. To date, a number of ER-targeting conjugates have been reported which attempt to exploit the high affinity and receptor selectivity of estrogen receptor ligands to deliver cytotoxic drugs to tumour cells [14,15,16,17,18]. In our investigation, antagonistic ER-ligands are key structural components utilised as the conjugate’s targeting mechanism. In the present study, the ER-targeting antagonist endoxifen is linked via a covalent amide bond to a Combretastatin A-4 analogue—itself a possible antimitotic agent. We now investigate if the introduction of steric hindrance provided by the Combretastatin CA-4 amide fragment, would enhance the ER antagonistic effects of the endoxifen conjugate in the ER positive MCF-7 cells, possibly by interferance with Helix-12. It is hypothesised that the combination of an antagonistic ER-ligand and the Combretastatin CA-4 related acrylic acid antimitotic agent may produce a selective antiproliferative action on ER-dependent cancers.

## 2. Experimental Section

### 2.1. Chemistry

All reagents were commercially available and were used without further purification unless otherwise indicated [19]. Tetrahydrofuran (THF) was distilled immediately prior to use from Na/Benzophenone under a slight positive pressure of nitrogen, toluene was dried by distillation from sodium and stored on activated molecular sieves (4 Å) and dichloromethane was dried by distillation from calcium hydride prior to use. Uncorrected melting points were measured on a Gallenkamp apparatus. Infra-red (IR) spectra were recorded as thin film on NaCl plates, or as potassium bromide discs on a Perkin Elmer FT-IR Specrtum 100 spectrometer (Perkin Elmer, Waltham, MA, USA). ^1^H, ^13^C and ^19^F nuclear magnetic resonance (NMR) spectra were recorded at 27 °C on a Brucker Avance DPX 400 spectrometer (400.13 MHz, ^1^H; 100.61 MHz, ^13^C; 376.47 MHz, ^19^F) (Brucker, Billerica, MA, USA) at 20 °C in either CDCl_3_ (internal standard tetramethylsilane (TMS)) or CD_3_OD by Dr. John O’Brien and Dr. Manuel Ruether in the School of Chemistry, Trinity College Dublin. For CDCl_3_, ^1^H-NMR spectra were assigned relative to the TMS peak at 0.00 δ and ^13^C-NMR spectra were assigned relative to the middle CDCl_3_ triplet at 77.00 ppm. For CD_3_OD, ^1^H and ^13^C-NMR spectra were assigned relative to the centre peaks of the CD_3_OD multiplets at 3.30 δ and 49.00 ppm respectively. ^19^F-NMR spectra were not calibrated. Electrospray ionisation mass spectrometry (ESI-MS) was performed in the positive ion mode on a liquid chromatography time-of-flight (TOF) mass spectrometer (Micromass LCT, Waters Ltd., Manchester, UK), equipped with electrospray ionization (ES) interface operated in the positive ion mode at the High Resolution Mass Spectrometry Laboratory by Dr. Martin Feeney in the School of Chemistry, Trinity College and a Micromass spectrometer (E.I. Mode) by Dr. Dilip Rai at the Centre for Synthesis and Chemical Biology, University College Dublin. Mass measurement accuracies of <±5 ppm were obtained. Low resolution mass spectra (LRMS) were acquired on a Hewlett-Packard 5973 MSD GC-MS system (Hewlett-Packard, Palo Alto, CA, USA) in electron impact (EI) mode. R_f_ values are quoted for thin layer chromatography on silica gel Merck F-254 plates, unless otherwise stated. Compounds were visually detected with UV at 254 and 366 nm. Flash column chromatography was carried out on Merck Kieselgel 60 (particle size 0.040–0.063 mm), Aldrich aluminium oxide, (activated, neutral, Brockmann I, 50 mesh) or Aldrich aluminium oxide, (activated, acidic, Brockmann I, 50 mesh). All products isolated were homogenous on TLC. Analytical high-performance liquid chromatography (HPLC) to determine the purity of the final compounds was performed using a Waters 2487 Dual Wavelength Absorbance detector, a Waters 1525 binary HPLC pump, a Waters In-Line Degasser AF and a Waters 717 plus Autosampler (Waters Corporation, Milford, MA, USA). The column used was a Varian Pursuit XRs C18 reverse phase 150 × 4.6 mm chromatography column (Agilent, Santa Clara, CA, USA). Samples were detected using a wavelength of 254 nm. All samples were analyzed using acetonitrile (70%): water (30%) over 10 min and a flow rate of 1 mL/min. Combretastatin A-4 (CA4) **26** was prepared as previously reported [20]. The acrylic acids **13** [21], **24** [21], **15** [22], **16** [23], **21** [22], **23** [24], **14** [25] and **25** [26] were prepared as previously reported.

#### 2.1.1. 4-{1-[4-(tert-Butyldimethylsilanyloxy)phenyl]-2-phenylbut-1-enylphenol **5**

Zinc dust (5.85 g, 90.0 mmol) was weighed out and transferred to a three-necked round-bottomed flask containing dry THF (100 mL). Titanium tetrachloride (8.55 g, 4.94 mL, 45 mmol), was carefully added via syringe to the mixture and then refluxed for 2 h under darkness and a nitrogen environment. The benzophenone **2** [27] (3.285 g, 10 mmol) and propiophenone **4a** (4.03 g, 4.00 mL, 30 mmol), were dissolved in dry THF (40 mL). This mixture was carefully added to the refluxing mixture in the round-bottomed flask via syringe. The mixture was then refluxed for a further 3 h. Afterwards, the mixture was allowed to cool then diluted with ethyl acetate (150 mL) and washed with 10% potassium carbonate solution (60 mL). After filtration, the organic layer was separated out and the aqueous layer was extracted with ethyl acetate (100 mL × 3). The combined organic layers were washed with 10% potassium carbonate solution (40 mL), water (50 mL) and brine (50 mL) then dried over anhydrous sodium sulfate, filtered and evaporated to dryness in vacuo to yield crude product. The material was purified via flash chromatography on silica gel (hexane:diethyl ether = 6:1) to afford an isomeric product mixture **5** (4.01 g, 93%, *E/Z* = 1.2:1) as a brown oil. ^1^H-NMR (400 MHz, CDCl_3_): δ 0.13 (s, 0.51 × 6H, SiCH_3_), 0.25 (s, 0.49 × 6H, SiCH_3_), 0.94–1.03 (m, 12H, SiC(CH_3_)_3_, CH_3_), 2.49–2.54 (q, 2H, *J* = 7.6 Hz, CH_2_), 4.78 (bs, 0.5H, OH), 5.05 (bs, 0.5H, OH), 6.49–6.52 (m, 2H, ArH), 6.68 (t, 2H, *J* = 8.5 Hz, ArH), 6.83–6.85 (m, 2H, ArH), 7.11–7.19 (m, 7H, ArH). ^13^C-NMR (100 MHz, CDCl_3_): δ −4.92, −4.82, −4.80, 13.17, 13.20, 17.74, 25.23, 27.21, 113.74, 114.44, 118.50, 119.08, 125.40, 126.21, 126.41, 126.68, 126.93, 127.19, 127.29, 127.37, 127.37, 129.26, 130.08, 130.35, 131.38, 131.70, 135.62, 135.94, 140.64, 142.11, 152.84, 153.05, 153.68. IR: ν_max_ (KBr) cm^−1^: 3560.4, 2967.6, 1738.9, 1598.4, 1463.1, 1445.1, 1251.1, 1115.8, 1072.3, 896.1, 739.1, 703.2, 655.0. HRMS (EI): Found 453.2220 (M + Na)^+^, C_28_H_34_O_2_NaSi requires 453.2226.

#### 2.1.2. 4-{1,2-Bis-[4-(tert-butyldimethylsilanyloxy)phenyl]but-1-enyl}phenol **6**

According to the general McMurry reaction method above with zinc dust (4.91 g, 75.1 mmol), titanium tetrachloride (7.12 g, 4.12 mL, 37.5 mmol), the benzophenone **2** [27] (2.74 g, 8.34 mmol) and the silylated propiophenone **4b** [28] (6.62 g, 25.0 mmol), the isomeric product mixture **6** was afforded (4.44 g, 95%, *E/Z* = 1.4:1) as a brown oil. ^1^H-NMR (400 MHz, CDCl_3_): δ 0.15 (s, 3H, CH_3_), 0.20 (s, 6H, CH_3_), 0.26 (s, 3H, CH_3_), 0.96–1.04 (m, 21H, CH_3_), 2.48 (q, 2H, *J* = 7.5 Hz, CH_2_), 6.50 (dd, 2H, *J* = 12.6 Hz, 8.6 Hz, ArH), 6.68 (t, 2H, *J* = 8.5 Hz, ArH), 6.74 (dd, 2H, *J* = 8.5 Hz, 5.0 Hz, ArH), 6.83 (dd, 2H, *J* = 11.5 Hz, 8.5 Hz, ArH), 6.98 (dd, 2H, *J* = 8.5 Hz, 4.0 Hz, ArH), 7.12 (dd, 2H, *J* = 8.6 Hz, 4.5 Hz, ArH), OH not observed. ^13^C-NMR (100 MHz, CDCl_3_): δ −4.88 (CH_3_), −4.88, −4.84, −4.80, 13.26, 17.75, 17.77, 17.81, 17.84, 25.23, 25.26, 25.29. 25.40, 28.34, 28.46, 113.72, 114.46, 118.43, 119.05, 119.10, 119.16, 130.15, 130.25, 130.37, 131.49, 131.73, 135.11, 135.21, 135.72, 136.08, 136.23, 136.54, 136.87, 140.19, 140.22, 152.82, 152.92, 153.27, 153.69, 153.72. IR: ν_max_ (KBr) cm^−1^: 3400.3, 2957.5, 2930.6, 2858.5, 1604.3, 1507.6, 1255.2, 1167.5, 914.9, 838.6, 804.1, 780.6. HRMS (EI): Found 583.3015 (M + Na)^+^, C_34_H_48_O_3_NaSi_2_ requires 583.3040.

#### 2.1.3. (4-{1-[4-(2-Bromoethoxy)phenyl]-2-phenylbut-1-enyl}phenoxy)-tert-butyldimethylsilane **7**

The phenolic triarylethylene **5** (3.16 g, 7.34 mmol), was dissolved in 1,2-dibromoethane (69.0 g, 32.0 mL, 367 mmol), with stirring. Tetrabutylammonium hydrogen sulfate (2.24 g, 6.61 mmol) was added, followed by 1 M sodium hydroxide solution (30 mL). The biphasic mixture was stirred vigorously at room temperature for 16 h. The reaction mixture was worked up via the addition of dichloromethane (100 mL) and sodium bicarbonate solution (100 mL). The aqueous layer was extracted with dichloromethane (100 mL). The organic layers were combined, dried over sodium sulfate and concentrated under in vacuo to yield crude product. The material was purified via flash chromatography on silica gel (hexane:diethyl ether = 40:1) to afford the product **7** (2.05 g, 52%, *E/Z* = 1.2:1) as a yellow oil. ^1^H-NMR (400 MHz, CDCl_3_): δ 0.12 (s, 0.55 × 6H, SiCH_3_), 0.25 (s, 0.45 × 6H, SiCH_3_), 0.94–1.02 (m, 12H, SiC(CH_3_)_3_, CH_3_), 2.47–2.53 (m, 2H, CH_2_), 3.58 (t, 0.45 × 2H, *J* = 6.0 Hz, NCH_2_), 3.69 (t, 0.55 × 2H, *J* = 6.5 Hz, NCH_2_), 4.18 (t, 0.45 × 2H, *J* = 6.5 Hz, OCH_2_), 4.34 (t, 0.55 × 2H, *J* = 6.0 Hz, OCH_2_), 6.49–6.58 (m, 2H, ArH), 6.71–6.93 (m, 4H, ArH), 7.09–7.21 (m, 7H, ArH). ^13^C-NMR (100 MHz, CDCl_3_): δ −4.92, −4.81, 13.16, 13.21, 13.70, 25.21, 25.23, 28.45, 28.73, 28.79, 31.15, 67.35, 113.00, 113.76, 118.52, 119.10, 125.48, 127.30, 127.40, 129.25, 130.09, 130.26, 131.37, 131.61, 135.88, 136.56, 140.74, 142.04, 155.45, 155.45, 156.28. IR: ν_max_ (KBr) cm^−1^: 3436.4, 2957.9, 2930.4, 2858.5, 1604.9, 1507.3, 1472.3, 1254.7, 1168.3, 916.2, 838.8, 804.2, 780.3. HRMS (EI): Found 559.1630 (M + Na)^+^, C_30_H_37_O_2_BrSiNa requires 559.1644.

#### 2.1.4. [2-(4-{1,2-Bis-[4-(tert-butyldimethylsilanyloxy)phenyl]but-1-enyl}phenoxy)ethyl] bromide **8**

According to the general alkylation method above with the phenol **6** (4.78 g, 8.51 mmol), 1,2-dibromoethane (80.0 g, 37.0 mL, 426 mmol), tetrabutylammonium hydrogen sulfate (2.60 g, 7.66 mmol) and 1 M sodium hydroxide solution (30 mL), the product **8** was afforded (3.07 g, 54%, *E/Z* = 1.4:1) as a yellow oil. ^1^H-NMR (400 MHz, CDCl_3_): δ 0.20–0.30 (m, 12H, CH_3_), 0.99–1.08 (m, 21H, CH_3_), 2.52–2.54 (m, 2H, CH_2_), 3.60 (t, 0.41 × 2H, *J* = 6.3 Hz, CH_2_), 3.68 (t, 0.59 × 2H, *J* = 6.0 Hz, CH_2_), 4.21 (t, 0.41 × 2H, *J* = 6.5 Hz, CH_2_), 4.34 (t, 0.59 × 2H, *J* = 6.3 Hz, CH_2_), 6.55–7.24 (m, 12H, ArH). ^13^C-NMR (100 MHz, CDCl_3_): δ −4.80, −4.77, 13.33, 13.36, 17.79, 17.85, 17.88, 25.31, 25.33, 25.36, 28.41, 28.54, 28.76, 28.83, 67.19, 67.37, 76.36, 76.68, 77.00, 130.19, 130.29, 130.35, 131.53, 131.70, 135.05, 135.15, 136.16, 136.41, 136.46, 136.78, 136.88, 140.38, 140.46, 153.03, 153.39, 153.82, 155.43, 156.28. IR: ν_max_ (KBr) cm^−1^: 3436.4, 2957.9, 2930.4, 2858.5, 1604.9, 1507.3, 1472.3, 1254.7, 1168.3, 916.2, 838.8, 804.2, 780.3. HRMS (EI): Found 689.2450 (M + Na)^+^, C_36_H_51_O_3_BrSi_2_Na requires 689.2458.

#### 2.1.5. [2-(4-{1-[4-(tert-Butyldimethylsilanyloxy)phenyl]-2-phenylbut-1-enyl}phenoxy)ethyl]methylamine **9**

Methylamine (in a 20 molar equivalent excess), was dissolved in anhydrous tetrahydrofuran (20 mL) together with the bromide **7** (0.54 g, 1.00 mmol) and sealed in a high-pressure tube. The reaction is heated to 60 °C while stirring for 48–72 h. After this time the reaction vessel was cooled. The reaction is worked up via the addition of a sodium carbonate/sodium hydrogencarbonate pH 10 buffer solution (50 mL) and the organics were extracted with dichloromethane (3 × 50 mL). The organic phases were combined, dried over sodium sulfate and the solvent evaporated in vacuo to afford a crude product, which is then purified via flash chromatography (dichloromethane:methanol) to afford the product **9** as a brown oil (0.38 g, 78%, *E/Z* = 1.3:1) [16]. ^1^H-NMR (400 MHz, CDCl_3_): δ 0.13 (s, 0.57 × 6H, Si(CH_3_)_2_), 0.26 (s, 0.43 × 6H, Si(CH_3_)_2_), 0.95–1.03 (m, 12H, (CH_3_)_3_), 2.45–2.53 (m, 5H, NCH_3_, CH_2_), 2.89 (s, 0.43 × 2H, CH_2_), 2.99 (s, 0.57 × 2H, CH_2_), 3.36 (s, 1H, NH), 3.95 (t, 0.43 × 2H, *J* = 5.0 Hz, CH_2_), 4.11 (t, 0.43 × 2H, *J* = 5.0 Hz, CH_2_), 6.50–7.20 (m, 13H, ArH). ^13^C-NMR (100 MHz, CDCl_3_): δ −4.91, −4.80, 13.21, 13.24, 17.75, 25.24, 25.26, 28.47, 28.60, 35.39, 35.26, 49.47, 50.03, 50.12, 65.73, 65.99, 112.80, 113.54, 118.54, 119.12, 125.44, 125.48, 127.33, 127.41, 129.25, 130.12, 130.20, 131.43, 131.57, 135.68, 136.02, 136.06, 136.35, 137.44, 137.53, 140.59, 140.65, 142.11, 142.19, 153.06, 153.84, 156.10, 156.94. IR: ν_max_ (KBr) cm^−1^: 3340.9, 2956.9, 2930.0, 2857.0, 1605.5, 1507.3, 1462.7, 1253.0, 1170.6, 1100.7, 1044.8, 914.4, 836.2, 805.0, 779.9, 700.0. HRMS (EI): Found 488.2980 (M + H)^+^, C_31_H_42_NO_2_Si requires 488.2985.

#### 2.1.6. [2-(4-{1,2-Bis-[4-(tert-butyldimethylsilanyloxy)phenyl]but-1-enyl}phenoxy)ethyl]methylamine **10**

According to the general amination method above with the bromide **8** (0.67 g, 1.00 mmol) and methylamine (in a 20 molar equivalent excess), the product **10** was afforded as a brown oil (0.47 g, 76%, *E/Z* = 1.4:1). ^1^H-NMR (400 MHz, CDCl_3_): δ 0.14 (s, 6H, CH_3_), 0.19 (s, 6H, CH_3_), 0.95–1.00 (m, 21H, CH_3_), 2.48 (q, 2H, *J* = 7.0 Hz, CH_2_), 2.55 (s, 3H, CH_3_), 2.80 (s, 1H, NH), 3.03 (s, 2H, NCH_2_), 4.13 (s, 2H, OCH_2_), 6.50 (d, 2H, *J* = 8.0 Hz, ArH), 6.66 (d, 2H, *J* = 6.5 Hz, ArH), 6.72 (d, 2H, *J* = 8.0 Hz, ArH), 6.90 (d, 2H, *J* = 7.0 Hz, ArH), 6.97 (d, 2H, *J* = 8.0 Hz, ArH), 7.17 (d, 2H, *J* = 7.0 Hz, ArH). ^13^C-NMR (100 MHz, CDCl_3_): δ −4.88, −4.84, 13.29, 17.74, 17.80, 25.24, 25.29, 28.35, 35.61, 50.22, 66.18, 113.51, 118.40, 119.03, 130.22, 130.25, 131.48, 135.06, 136.19, 136.24, 136.94, 140.22, 152.94, 153.30, 156.91. IR: ν_max_ (KBr) cm^−1^: 3401.3, 2956.7, 2930.4, 2857.6, 1606.1, 1508.0, 1471.8, 1253.9, 1169.7, 915.2, 835.6, 804.7, 779.5. HRMS (EI): Found 618.3785 (M + H)^+^, C_37_H_56_NO_3_Si_2_ requires 618.3799.

#### 2.1.7. 4-{1-[4-(2-Methylaminoethoxy)phenyl]-2-phenylbut-1-enyl}phenol **11** (Endoxifen)

The silyl ether amine **9** (0.12 g, 0.25 mmol), was dissolved in a minimum amount (~5 mL) of THF while stirred under nitrogen. An equimolar quantity of TBAF was added, relative to the number of silyl protecting groups present and the mixture was allowed stir for 16–24 h. The reaction was monitored via TLC (dichloromethane:methanol). The solvent was evaporated to dryness. The residue was redissolved in dichloromethane (~30 mL) and then washed with a quantity of 10% HCl solution (~20 mL). The organic phase was dried over sodium sulphate and evaporated to dryness in vacuo. The residue was purified via flash chromatography on silica gel (DCM:MeOH) to afford the product as an isomeric mixture of product **11** [29] as a brown oil (85 mg, 93%, *E*/*Z* = 1.1:1). ^1^H-NMR (400 MHz, CDCl_3_): δ 0.95 (t, 3H, *J* = 7.5 Hz, CH_3_), 2.48–2.56 (m, 6H, NCH_3_, CH_3_), 2.94 (s, 0.52 × 2H, CH_2_), 3.04 (s, 0.48 × 2H, CH_2_), 3.96 (t, 0.52 × 2H, *J* = 4.8 Hz, CH_2_), 4.12 (t, 0.48 × 2H, *J* = 4.8 Hz, CH_2_), 4.92 (s, 2H, NH, OH), 6.43–7.20 (m, 13H, ArH). ^13^C-NMR (100 MHz, CDCl_3_): δ 13.25, 28.56, 28.63, 49.42, 57.90, 64.66, 112.81, 113.58, 114.12, 114.82, 125.42, 127.36, 127.39, 129.27, 130.17, 130.20, 131.54, 131.58, 134.32, 134.67, 136.07, 136.52, 137.48, 140.20, 140.41, 142.23, 142.26, 154.29, 155.22, 155.62, 156.49. IR: ν_max_ (KBr) cm^−1^: 3391.6, 3188.4, 2956.7, 2929.8, 2870.4, 1606.2, 1507.7 (C=C), 1462.0, 1238.8, 1170.4, 1036.6, 835.9, 770.8, 699.9. HRMS (EI): Found 374.2116 (M + H)^+^, C_25_H_28_NO_2_ requires 374.2120.

#### 2.1.8. 4-(1-(4-(2-(Methylamino)ethoxy)phenyl)-2-(4-hydroxyphenyl)but-1-enyl)phenol **12**

According to the silyl ether deprotection method above with amine **10** (0.15 g, 0.24 mmol), an isomeric mixture of product **12** was afforded as a brown oil (87 mg, 92%, *E/Z* = 1:1.2). ^1^H-NMR (400 MHz, CDCl_3_): δ 0.89–0.94 (m, 6H, CH_3_), 2.39–2.49 (m, 2H, CH_2_), 2.65–2.70 (m, 3H, NCH_3_), 3.21–3.34 (m, 3H, OH, NCH_2_), 4.08–4.25 (m, 2H, OCH_2_), 6.42–7.14 (m, 12H, ArH). ^13^C-NMR (100 MHz, CDCl_3_): δ 12.21, 27.90, 27.97, 32.65, 32.71, 48.23, 48.30, 63.28, 63.62, 112.60, 113.17, 113.26, 113.30, 113.41, 113.85, 113.98, 114.01, 129.75, 129.87, 130.07, 130.23, 131.19, 131.22, 131.28, 133.06, 134.71, 136.76, 136.90, 137.01, 139.88, 140.22, 154.75, 154.78, 155.29, 155.51. IR: ν_max_ (KBr) cm^−1^: 3391.3, 3289.9, 2960.8, 2927.5, 2870.1, 1607.7, 1509.4, 1461.6, 1371.3, 1236.1, 1169.8, 1102.2, 1035.4, 832.0. HRMS (EI): Found 390.2057 (M + H)^+^, C_25_H_28_NO_3_ requires 386.2069.

#### 2.1.9. (*E*)-3-(3-Amino-4-methoxyphenyl)-2-(3,4,5-trimethoxyphenyl)acrylic acid **16**

The nitro compound **21** [22], (0.40 g, 1.02 mmol) was dissolved in 19.0 mL glacial acetic acid while zinc powder (3.84 g, 0.06 mmol) was added to the mixture. The reaction was stirred at room temperature for 3 h, then filtered through Celite. The filtrate was diluted with DCM (50 mL) and washed with 1 M sodium hydroxide solution (3 × 250 mL). The organic layer was dried over sodium sulfate and evaporated to dryness to afford yellow crystals **16** (0.25 g, 67%, m.p. 199–202 °C) [23]. ^1^H-NMR (400 MHz, *d*-DMSO): δ 3.69–3.72 (m, 12H, OCH_3_), 3.78 (t, 2H, NH_2_, *J* = 9.3 Hz), 6.28 (d, 1H, *J* = 8.5 Hz), 6.44 (s, 2H), 6.51 (s, 1H), 6.66 (d, 1H, *J =* 8.5 Hz), 6.75–6.79 (m, 1H), 7.54 (s, 1H, C=CH). ^13^C-NMR (100 MHz, *d*-DMSO): δ 55.27 (OCH_3_), 55.91 (OCH_3_), 60.10 (OCH_3_), 103.11, 106.77, 110.09, 116.20, 119.19, 126.87, 129.94, 132.38, 136.85, 137.17, 139.72, 147.39, 152.87, 152.99 (COOH). IR: ν_max_ (KBr) cm^−1^: 3437.06 (w), 3357.48, 2939.23, 1672.37, 1587.20, 1505.99, 1439.78, 1411.72, 1268.86, 1238.95, 1171.02, 1123.26 (s), 1028.15. HRMS (EI): Found 360.1394 (M + H)^+^, C_19_H_22_NO_6_ requires 360.1369.

#### 2.1.10. General Method for Synthesis of Acrylic Acids **17**–**20**, **22**

A mixture of the appropriate benzaldehyde (1 equivalent), the appropriate phenylacetic acid (~0.50 g, 1 equivalent), acetic anhydride (2 mL) and triethylamine (1 mL) were heated under reflux for 3 h. After acidification with concentrated hydrochloric acid (~5 mL), the resulting solid was filtered off and recrystallised to yield the appropriate acrylic acid.

#### 2.1.11. (*E*)-3-(4-Bromophenyl)-2-(3,4,5-trimethoxyphenyl)acrylic Acid **17**

4-Bromobenzaldehyde (0.41 g, 2.21 mmol) and 3,4,5-trimethoxyphenylacetic acid (0.50 g, 2.21 mmol) were reacted following the general method above. Recrystallisation from ethanol yielded the acrylic acid **17** as fine yellow needles (0.36 g, 41%, m.p. 227–230 °C) [22]. ^1^H-NMR (400 MHz, *d*-DMSO): δ 3.67 (s, 6H, OCH_3_), 3.71 (s, 3H, OCH_3_), 6.45 (s, 2H, ArH), 7.06 (d, 2H, *J* = 8.5Hz), 7.46 (d, 2H, *J* = 8.5 Hz), 7.69 (s, 1H, C=CH), 12.76 (s, 1H, COOH). ^13^C-NMR (100 MHz, *d*-DMSO): δ 55.94 (OCH_3_), 60.15 (OCH_3_), 106.60, 122.46 (C-Br), 131.32, 133.73, 134.07, 137.13, 137.54, 153.11, 168.15 (COOH). IR: ν_max_ (KBr) cm^−1^: 3435.94 (w), 2936.34, 1667.10, 1582.78, 1505.97, 1465.88, 1453.56, 1411.97, 1309.07, 1287.21, 1240.39, 1132.43, 1008.37. HRMS (EI): Found 415.0170 (M + Na)^+^, C_18_H_17_O_5_NaBr requires 415.0157.

#### 2.1.12. (*E*)-3-(4-Fluorophenyl)-2-(3,4,5-trimethoxyphenyl)acrylic acid **18**

4-Fluorobenzaldehyde (0.27 g, 2.21 mmol) and 3,4,5-trimethoxyphenylacetic acid (0.50 g, 2.21 mmol) were reacted following the general method above. Recrystallisation from ethanol yielded the acrylic acid **18** as fine yellow needles (0.43 g, 59%, m.p. 211–213 °C). ^1^H-NMR (400 MHz, *d*-DMSO): δ 3.67 (s, 6H, OCH_3_), 3.71 (s, 3H, OCH_3_), 6.46 (s, 2H, ArH), 7.08–7.19 (m, 4H, ArH), 7.73 (s, 1H, C=CH), 12.52 (s, 1H, COOH). ^13^C-NMR (100 MHz, *d*-DMSO): δ 55.91 (OCH_3_), 60.13 (OCH_3_), 106.59, 115.24, 115.46, 131.01, 131.53, 132.39, 132.48, 132.98, 137.02, 137.64, 153.16, 168.27 (COOH). ^19^F-NMR (100 MHz, *d*-DMSO): δ −111.64 IR: ν_max_ (KBr) cm^−1^: 3436.06 (w), 2942.89, 2832.08, 1665.92, 1597.65, 1582.99, 1507.68, 1458.71, 1411.77, 1306.89, 1294.76, 1240.29, 1219.25, 1130.42, 1005.88, 837.04. HRMS (EI): Found 355.0989 (M + Na)^+^, C_18_H_17_O_5_FNa requires 355.0958.

#### 2.1.13. (*E*)-3-p-Tolyl-2-(3,4,5-trimethoxyphenyl)acrylic acid **19**

*p*-Tolualdehyde (0.27 g, 2.21 mmol) and 3,4,5-trimethoxyphenylacetic acid (0.50 g, 2.21 mmol) were reacted following the general method above. Recrystallisation from ethanol yielded the acrylic acid **19** as fine yellow needles (0.27 g, 37%, m.p. 190–193 °C). ^1^H-NMR (400 MHz, *d*-DMSO): δ 2.24 (s, 3H, CH_3_), 3.67 (s, 6H, OCH_3_), 3.71 (s, 3H, OCH_3_), 6.45 (s, 2H, ArH), 7.03 (2xd, 4H, 8 Hz, ArH), 7.69 (s, 1H, C=CH), 12.61 (s, 1H, COOH). ^13^C-NMR (100 MHz, *d*-DMSO): δ 20.87 (CH_3_), 55.92 (OCH_3_), 60.14 (OCH_3_), 106.63, 128.96, 130.26, 131.56, 132.01, 132.22, 136.95, 138.91, 139.04, 153.09, 168.40 (COOH). IR: ν_max_ (KBr) cm^−1^: 3436.54 (w), 2939.20, 1670.98 (s), 1581.01, 1505.34, 1412.52, 1294.34, 1241.50, 1185.30, 1127.19 (s), 1000.57. HRMS (EI): Found 351.1198 (M + Na)^+^, C_19_H_20_O_5_Na requires 351.1208.

#### 2.1.14. (*E*)-3-(4-Methylsulfanylphenyl)-2-(3,4,5-trimethoxyphenyl)acrylic acid **20**

4-Methylthiobenzaldehyde (0.34 g, 2.21 mmol), 3,4,5-trimethoxyphenylacetic acid (0.50 g, 2.21 mmol) reacted following the general method above. Recrystallisation from ethanol yielded the acrylic acid **20** as fine yellow needles (0.38 g, 48%, m.p. 194–196 °C). ^1^H-NMR (400 MHz, *d*-DMSO): δ 2.43 (s, 3H, SCH_3_), 3.68 (s, 6H, OCH_3_), 3.72 (s, 3H, OCH_3_), 6.47 (s, 2H, ArH), 7.08 (2xd, 4H, *J* = 8.5 Hz, ArH), 7.68 (s, 1H, C=CH), 12.64 (s, 1H, COOH). ^13^C-NMR (100 MHz, *d*-DMSO): δ 14.01 (SCH_3_), 55.93 (OCH_3_), 60.15 (OCH_3_), 106.50, 125.02, 130.56, 130.71, 132.01, 132.13, 136.95, 138.43, 140.36, 153.17, 168.36 (COOH). IR: ν_max_ (KBr) cm^−1^: 3435.88 (w), 2939.66, 1667.30, 1587.76, 1506.19, 1410.67, 1286.01, 1240.91, 1125.30, 1087.58, 1001.09. HRMS (EI): Found 383.0940 (M + Na)^+^, C_19_H_20_O_5_NaS requires 383.0929.

#### 2.1.15. (*E*)-3-Naphthalen-2-yl-2-(3,4,5-trimethoxyphenyl)acrylic acid **22**

β-Naphthaldehyde (0.35 g, 2.21 mmol), 3,4,5-trimethoxyphenylacetic acid (0.50 g, 2.21 mmol) were reacted following the general method above. Recrystallisation from ethanol yielded the acrylic acid **22** as fine yellow needles (0.41 g, 51%, m.p. 238–240 °C). ^1^H-NMR (400 MHz, *d*-DMSO): δ 3.65 (s, 6H, OCH_3_), 3.73 (s, 3H, OCH_3_), 6.52 (s, 2H, ArH), 7.02 (dd, 1H, *J* = 1.5 Hz, 8.5 Hz, ArH), 7.50 (d, 2H, *J* = 2 Hz, ArH), 7.52–7.90 (m, 6H ArH, CH=), 12.73 (s, 1H, COOH). ^13^C-NMR (100 MHz, *d*-DMSO): δ 55.97 (OCH_3_), 60.20 (OCH_3_), 106.99, 126.18, 126.56, 127.18, 127.40, 127.46, 128.25, 131.36, 131.75, 132.16, 132.58, 132.80, 133.43, 137.20, 138.88, 153.08, 168.34 (COOH). IR: ν_max_ (KBr) cm^−1^: 3435.50, 2937.78, 1664.26, 1581.88, 1504.78, 1454.14, 1411.09, 1287.35, 1239.16, 1127.39, 1005.19. HRMS (EI): Found 387.1222 (M + Na)^+^, C_22_H_20_O_5_Na requires 387.1208.

#### 2.1.16. General Method for Synthesis of Endoxifen-Acrylic Acid Conjugates **27**–**46**

A mixture of the required acrylic acid (1 equivalent (eq.), 0.15 mmol), DCC (1 eq., 0.15 mmol, 0.03 g) and HOBt (1 eq., 0.15 mmol, 0.02 g) were suspended in 3 mL of anhydrous DCM and stirred for 10 min under a nitrogen atmosphere. The required silyl-protected endoxifen analogue, **9** (0.08 g, 0.15 mmol, 1 eq.) or **10** (0.10 g, 0.15 mmol, 1 eq.), was dissolved in 3 mL of anhydrous DCM and slowly added to the mixture via syringe. Reaction was allowed stir for 24–48 h. Reaction was monitored via TLC (DCM:MeOH, 4:1). The reaction mixture was diluted to 15 mL with anhydrous DCM and filtered to remove DCU. The filtrate was evaporated to dryness under reduced pressure. The residue was dissolved in 3 mL anhydrous THF and stirred under a nitrogen atmosphere. A solution of 0.1 M TBAF (2 equivalents) was added to the mixture and allowed stir for 24 h. The mixture was evaporated to dryness under reduced pressure. The residue was dissolved in DCM and washed with 10% HCl solution. The resulting organic phase was dried over sodium sulfate and evaporated to dryness under vacuum. The residue was purified via flash chromatography on silica gel (DCM:MeOH, 20:1) to yield a *E*/*Z* isomeric mixture of the products.

#### 2.1.17. (*E*)-3-(3-Hydroxy-4-methoxyphenyl)-*N*-(2-{4-[(*E/Z*)-1-(4-hydroxyphenyl)-2-phenylbut-1-enyl]phenoxy}ethyl)-*N*-methyl-2-(3,4,5-trimethoxyphenyl) acrylamide **27**

The acrylic acid analogue **13** was reacted with the endoxifen derivative **9**, following the general method above. The product **27** was afforded as a brown oil (103 mg, 94%), then changed to a semi-solid resin. ^1^H-NMR (400 Hz, CDCl_3_): δ 0.90–0.96 (m, 3H, CH_3_), 2.47–2.52 (m, 3H, CH_3_), 3.04–3.24 (m, 3H, NCH_3_), 3.44–4.35 (m, 16H, OCH_3_, CH_2,_, NCH_2_, OCH_2_), 6.40–7.20 (m, 19H, ArH), OH not observed. ^13^C-NMR (100 MHz, CDCl_3_): δ 13.17, 13.20, 24.45, 25.12, 28.62, 33.44, 48.72, 55.44, 55.57, 55.67, 60.50, 65.71, 105.58, 109.76, 112.65, 113.40, 113.93, 114.64, 115.01, 121.52, 125.46, 127.37, 127.39, 128.07, 129.25, 129.58, 130.17, 130.23, 131.55, 131.59, 135.20, 137.33, 140.44, 142.12, 144.68, 145.96, 145.98, 152.83, 153.72, 154.66, 156.62. IR: ν_max_ (KBr) cm^−1^: 3420.3, 3376.8, 3270.5, 2930.8, 2850.2, 1626.4, 1606.8, 1580.6, 1507.9, 1462.9, 1410.8, 1274.7, 1238.4, 1169.3, 1126.3, 1026.8, 901.1, 834.9, 762.8, 701.4. HRMS (EI): Found 738.3043 (M + Na)^+^, C_44_H_45_NO_8_Na requires 738.3043.

#### 2.1.18. (*E*)-*N*-(2-{4-[(*E/Z*)-1,2-Bis-(4-hydroxyphenyl)but-1-enyl]-phenoxy}ethyl)-3-(3-hydroxy-4-methoxyphenyl)-*N*-methyl-2-(3,4,5-trimethoxyphenyl)acrylamide **28**

The acrylic acid analogue **13** was reacted with the endoxifen derivative **10**, following the general method above,. The product **28** was afforded as a brown oil (104 mg, 92%), then changes to a semi-solid resin. ^1^H-NMR (400 MHz, CDCl_3_): δ 0.90–0.96 (m, 3H, CH_3_), 2.40–2.50 (m, 3H, CH_3_), 3.04–3.25 (m, 3H, NCH_3_), 3.53–4.30 (m, 16H, OCH_3_, CH_2,_, NCH_2_, OCH_2_), 6.40–7.20 (m, 18H, ArH), 2xOH not observed. ^13^C-NMR (100 MHz, CDCl_3_): δ 13.28, 25.11, 33.42, 48.67, 55.46, 55.68, 60.55, 66.29, 105.32, 105.47, 112.61, 113.37, 113.99, 114.49, 114.53, 114.65, 128.44, 128.73, 128.88, 130.15, 130.29, 130.48, 131.52, 131.59, 133.59, 134.94, 137.49, 140.06, 152.72, 152.81, 153.12, 153.74, 154.17, 154.66, 156.88. IR: ν_max_ (KBr) cm^−1^: 3428.6, 3376.8, 3270.5, 2932.4, 2869.6, 1607.9, 1580.6, 1509.6, 1462.9, 1410.0, 1272.0, 1238.1, 1169.2, 1125.6, 1050.2, 1023.7, 901.0, 833.5. HRMS (EI): Found 732.3162 (M + H)^+^, C_44_H_46_NO_9_ requires 732.3173.

#### 2.1.19. (*E*)-*N*-(2-{4-[(*E/Z*)-1-(4-Hydroxyphenyl)-2-phenylbut-1-enyl]phenoxy}-ethyl)-3-(4-methoxyphenyl)-*N*-methyl-2-(3,4,5-trimethoxyphenyl)acrylamide **29**

The acrylic acid analogue **14** was reacted with the endoxifen derivative **9**, following the general method above. The product **29** was afforded as a brown oil (97 mg, 90%), then changes to a semi-solid resin. ^1^H-NMR (400 MHz, CDCl_3_): δ 0.91–0.95 (m, 3H, CH_3_), 2.48–2.50 (m, 2H, CH_2_), 3.06–3.21 (m, 3H, NCH_3_), 3.50–3.91 (m, 14H, OCH_3_, CH_2,_), 4.10 (m, 0.30 × 2H, OCH_2_), 4.26–4.33 (m, 0.70 × 2H, OCH_2_), 5.68 (s, 1H, OH), 6.51–7.17 (m, 19H, ArH). ^13^C-NMR (100 MHz, CDCl_3_): δ 13.17, 13.20, 24.45, 25.12, 28.55, 28.62, 33.44, 48.72, 53.00, 55.44, 55.57, 55.67, 60.50, 105.58, 109.76, 112.65, 113.40, 113.93, 114.64, 115.01, 121.52, 125.46, 127.37, 127.39, 128.07, 129.25, 129.58, 130.17, 130.23, 131.55, 131.59, 134.78, 135.20, 137.33, 140.44, 142.12, 144.68, 145.96, 145.98, 152.83, 153.72, 154.66, 156.62. IR: ν_max_ (KBr) cm^−1^: 3468.6, 3372.0, 3327.3, 2929.7, 2850.8, 1626.3, 1606.0, 1579.0, 1509.4, 1463.2, 1411.3, 1310.6, 1243.3, 1174.1, 1127.6, 1030.3, 829.4. HRMS (EI): Found 722.3073 (M + Na)^+^, C_44_H_45_NO_7_Na requires 722.3094.

#### 2.1.20. (*E*)-*N*-(2-{4-[(*E/Z*)-1,2-Bis-(4-hydroxyphenyl)but-1-enyl]phenoxy}ethyl)-3-(4-methoxyphenyl)-*N*-methyl-2-(3,4,5-trimethoxyphenyl)acrylamide **30**

The acrylic acid analogue **14** was reacted with the endoxifen derivative **10**, following the general method above. The product **30** was afforded as a brown oil (104 mg, 94%), then changes to a semi-solid resin. ^1^H-NMR (400 MHz, CDCl_3_): δ 0.91–0.94 (m, 3H, CH_3_), 2.42–2.46 (m, 2H, CH_2_), 3.06–3.22 (m, 3H, NCH_3_), 3.53–4.24 (m, 16H, OCH_3_, CH_2,_), 5.74–5.80 (m, 2H, OH), 6.42–7.12 (m, 18H, ArH). ^13^C-NMR (100 MHz, CDCl_3_): δ 13.26, 28.30, 28.40, 38.75, 47.38, 55.45, 55.50, 55.56, 60.54, 60.58, 65.87, 105.36, 105.54, 109.80, 112.63, 113.41, 113.93, 114.40, 114.57, 115.08, 121.54, 127.92, 129.84, 130.21, 130.37, 131.55, 133.78, 134.78, 135.11, 136.66, 144.54, 144.62, 146.05, 152.77, 152.87, 153.46, 153.79, 153.92, 154.24. IR: ν_max_ (KBr) cm^−1^: 3327.3, 2929.5, 2850.8, 1626.3, 1607.1, 1579.7, 1510.3, 1449.5, 1310.9, 1243.3, 1172.5, 1127.2, 1046.6, 892.6, 829.4. HRMS (EI): Found 738.3076 (M + Na)^+^, C_44_H_45_NO_8_Na requires 738.3043.

#### 2.1.21. (*E*)-*N*-(2-{4-[(*E/Z*)-1-(4-Hydroxyphenyl)-2-phenylbut-1-enyl]phenoxy}ethyl)-2-(4-methoxyphenyl)-*N*-methyl-3-(3,4,5-trimethoxyphenyl) acrylamide **31**

The acrylic acid analogue **23** was reacted with the endoxifen derivative **9**, following the general method above. The product **31** was afforded as a brown oil (99 mg, 92%), then changes to a semi-solid resin. ^1^H-NMR (400 MHz, CDCl_3_): δ 0.91–0.95 (m, 3H, CH_3_), 2.49–2.51 (m, 2H, CH_2_), 3.08 (s, 1.5H, NCH_3_), 3.17 (s, 1.5H, NCH_3_), 3.57–3.88 (m, 14H, OCH_3_, NCH_2,_), 4.09 (m, 0.35 × 2H, OCH_2_), 4.25 (m, 0.33 × 2H, OCH_2_), 4.39 (d, 0.32 × 2H, *J =* 8.0 Hz, OCH_2_), 6.39–7.31 (m, 20H, ArH), OH not observed. ^13^C-NMR (100 MHz, CDCl_3_): δ 13.15, 13.19, 24.45, 25.14, 33.44, 54.84, 55.33, 60.42, 106.20, 106.23, 113.72, 113.98, 114.69, 125.43, 127.36, 129.23, 129.91, 129.94, 130.12, 130.19, 130.41, 131.49, 131.52, 152.27, 152.30. IR: ν_max_ (KBr) cm^−1^: 3430.0, 3327.5, 2929.6, 2850.8, 1626.4, 1579.1, 1509.0, 1462.7, 1244.2, 1174.2, 1127.0, 835.5, 641.0. HRMS (EI): Found 722.3099 (M + Na)^+^, C_44_H_45_NO_7_Na requires 722.3094.

#### 2.1.22. (*E*)-*N*-(2-{4-[(*E/Z*)-1,2-Bis-(4-hydroxyphenyl)but-1-enyl]phenoxy}ethyl)-2-(4-methoxyphenyl)-*N*-methyl-3-(3,4,5-trimethoxyphenyl)acrylamide **32**

The acrylic acid analogue **23** was reacted with the endoxifen derivative **10**, following the general method above. The product **32** was afforded as a brown oil (99 mg, 90%), then changes to a semi-solid resin. ^1^H-NMR (400 MHz, CDCl_3_): δ 0.90–0.93 (m, 3H, CH_3_), 2.44–2.45 (m, 2H, CH_2_), 3.09 (s, 1.5H, NCH_3_), 3.16 (s, 1.5H, NCH_3_), 3.57–3.86 (m, 14H, OCH_3_, NCH_2,_), 4.08 (m, 0.31 × 2H, OCH_2_), 4.24 (m, 0.28 × 2H, OCH_2_), 4.39 (d, 0.41 × 2H, *J =* 8.0 Hz, OCH_2_), 6.39–7.28 (m, 19H, ArH), 2xOH not observed. ^13^C-NMR (100 MHz, CDCl_3_): δ 13.22, 24.43, 25.12, 25.20, 33.41, 55.34, 60.42, 106.25, 112.79, 113.51, 113.76, 113.98, 114.47, 114.66, 129.92, 130.14, 130.20, 130.29, 130.39, 130.78, 130.89, 131.54, 137.18, 152.27, 154.61, 156.82, 158.97. IR: ν_max_ (KBr) cm^−1^: 3327.7, 2929.5, 2850.7, 1625.8, 1579.3, 1510.5, 1449.9, 1244.1, 1171.7, 1126.7, 1033.0, 834.6, 641.1. HRMS (EI): Found 738.3065 (M + Na)^+^, C_44_H_45_NO_8_Na requires 738.3043.

#### 2.1.23. (*E*)-3-Benzo[1,3]dioxol-5-yl-*N*-(2-{4-[(*E/Z*)-1-(4-hydroxyphenyl)-2-phenylbut-1-enyl]phenoxy}ethyl)-2-(4-methoxyphenyl)-*N*-methylacrylamide **33**

The acrylic acid analogue **25** was reacted with the endoxifen derivative **9**, following the general method above. The product **33** was afforded as a brown oil (94 mg, 93%), then changes to a semi-solid resin. ^1^H-NMR (400 MHz, CDCl_3_): δ 0.92–0.96 (m, 3H, CH_3_), 2.49–2.51 (m, 2H, CH_2_), 3.04–3.13 (m, 3H, NCH_3_), 3.47–4.24 (m, 7H, OCH_3_, CH_2,_), 5.91 (s, 2H, O_2_CH_2_), 6.46–7.23 (m, 21H, ArH), OH not observed. ^13^C-NMR (100 MHz, CDCl_3_): δ 13.16, 13.19, 24.37, 25.05, 28.54, 28.60, 33.24, 48.97, 54.75, 100.57, 107.69, 108.61, 112.77, 113.52, 113.83, 113.96, 114.68, 123.61, 125.43, 127.36, 129.06, 129.25, 129.69, 129.73, 130.15, 130.20, 131.54, 140.42, 142.25, 146.71, 146.86, 146.89, 153.75, 154.71. IR: ν_max_ (KBr) cm^−1^: 3435.8, 3323.7, 2929.1, 2850.7, 1626.2, 1575.7, 1509.7, 1243.6, 1036.2, 835.0, 630.8. HRMS (EI): Found 676.2667 (M + Na)^+^, C_42_H_39_NO_6_Na requires 676.2675.

#### 2.1.24. (*E*)-3-Benzo[1,3]dioxol-5-yl-*N*-(2-{4-[(*E/Z*)-1,2-bis-(4-hydroxyphenyl)but-1-enyl]phenoxy}ethyl)-2-(4-methoxyphenyl)-*N*-methylacrylamide **34**

The acrylic acid analogue **25** was reacted with the endoxifen derivative **10**, following the general method above. The product **34** was afforded as a brown oil (95 mg, 92%), then changes to a semi-solid resin. ^1^H-NMR (400 MHz, CDCl_3_): δ 0.91–0.94 (m, 3H, CH_3_), 2.44–2.46 (m, 3H, CH_2_), 3.05–3.12 (m, 3H, NCH_3_), 3.48–4.53 (m, 7H, OCH_3_, CH_2,_), 5.90 (m, 2H, OCH_2_O), 6.45–7.26 (m, 20H, ArH), OH not observed. ^13^C-NMR (100 MHz, CDCl_3_): δ 13.26, 13.25, 24.42, 25.10, 28.42, 33.38, 48.76, 54.76, 54.80, 100.57, 107.73, 108.62, 109.21, 112.79, 113.52, 113.84, 113.99, 114.50, 114.66, 128.99, 129.73, 130.17, 130.22, 130.33, 130.51, 131.56, 140.04, 146.74, 146.84, 146.87, 154.53, 156.93. IR: ν_max_ (KBr) cm^−1^: 3412.2, 2929.5, 2850.2, 1606.9, 1510.6, 1488.3, 1444.2, 1242.9, 1172.0, 1035.9, 930.8, 833.3. HRMS (EI): Found 692.2648 (M + Na)^+^, C_42_H_39_NO_7_Na requires 692.2624.

#### 2.1.25. (*E*)-*N*-(2-{4-[(*E/Z*)-1-(4-Hydroxyphenyl)-2-phenylbut-1-enyl]phenoxy}ethyl)-*N*-methyl-3-naphthalen-2-yl-2-(3,4,5-trimethoxyphenyl) acrylamide **35**

The acrylic acid analogue **22** was reacted with the endoxifen derivative **9**, following the general method above. The product **35** was afforded as a brown oil (101 mg, 91%), then changes to a semi-solid resin. ^1^H-NMR (400 MHz, CDCl_3_): δ 0.91–0.95 (m, 3H, CH_3_), 2.48–2.50 (m, 2H, CH_2_), 3.11–3.27 (m, 3H, NCH_3_), 3.50–4.35 (m, 13H, OCH_3_, CH_2,_), 6.45–7.77 (m, 23H, ArH), OH not observed. ^13^C-NMR (100 MHz, CDCl_3_): δ 13.18, 13.23, 24.45, 25.13, 33.45, 48.72, 53.01, 55.48, 55.61, 60.52, 105.32, 112.65, 113.41, 113.95, 114.66, 125.46, 125.78, 125.80, 125.97, 126.27, 126.93, 126.95, 127.17, 127.37, 127.39, 127.57, 128.87, 129.24, 130.16, 130.27, 131.55, 131.62, 132.34, 132.73, 134.75, 135.09, 136.93, 137.33, 140.44, 140.59, 142.08, 152.85, 153.80, 154.75, 156.63, 171.43, 171.50. IR: ν_max_ (KBr) cm^−1^: 3425.1, 3327.0, 2929.1, 2850.6, 1625.9, 1579.0, 1507.3, 1449.4, 1410.0, 1310.4, 1242.0, 1170.2, 1127.2, 905.1, 833.8, 701.1, 641.0. HRMS (EI): Found 742.3143 (M + Na)^+^, C_47_H_45_NO_6_Na requires 742.3145.

#### 2.1.26. (*E*)-*N*-(2-{4-[(*E/Z*)-1,2-Bis-(4-hydroxyphenyl)but-1-enyl]phenoxy}ethyl)-*N*-methyl-3-naphthalen-2-yl-2-(3,4,5-trimethoxyphenyl)acrylamide **36**

The acrylic acid analogue **22** was reacted with the endoxifen derivative **10**, following the general method above. The product **36** was afforded as a brown oil (98 mg, 87%), then changes to a semi-solid resin. ^1^H-NMR (400 MHz, CDCl_3_): δ 0.90–0.92 (m, 3H, CH_3_), 2.42–2.44 (m, 2H, CH_2_), 3.11–3.26 (m, 3H, NCH_3_), 3.44–4.54 (m, 13H, OCH_3_, CH_2,_), 6.45–7.77 (m, 22H, ArH), 2xOH not observed. ^13^C-NMR (100 MHz, CDCl_3_): δ 13.26, 24.43, 25.09, 33.39, 48.75, 55.41, 55.47, 55.56, 60.53, 60.57, 105.43, 105.57, 106.40, 112.62, 113.38, 114.00, 114.49, 114.54, 114.65, 125.82, 126.01, 126.24, 126.52, 126.97, 127.08, 127.17, 127.58, 128.03, 128.83, 128.89, 129.59, 129.85, 130.17, 130.27, 130.32, 131.17, 131.54, 132.37, 132.73, 133.63, 136.52, 140.07, 152.79, 152.88, 152.96, 153.71, 154.06, 154.14, 154.61, 155.67, 156.93, 171.69. IR: ν_max_ (KBr) cm^−1^: 3430.0, 3327.7, 2929.5, 2850.7, 1626.0, 1578.4, 1507.4, 1311.2, 1243.1, 1126.8, 641.0. HRMS (EI): Found 758.3123 (M + Na)^+^, C_47_H_45_NO_7_Na requires 758.3094.

#### 2.1.27. (*E*)-*N*-(2-{4-[(*E/Z*)-1-(4-Hydroxyphenyl)-2-phenylbut-1-enyl]phenoxy}ethyl)-*N*-methyl-3-p-tolyl-2-(3,4,5-trimethoxyphenyl)acrylamide **37**

The acrylic acid analogue **19** was reacted with the endoxifen derivative **9**, following the general method above. The product **37** was afforded as a brown oil (98 mg, 93%), then changes to a semi-solid resin. ^1^H-NMR (400 MHz, CDCl_3_): δ 0.91–0.94 (m, 6H, CH_3_), 2.31 (s, 3H, CH_3_), 2.49–2.51 (m, 2H, CH_2_), 3.08–3.24 (m, 3H, NCH_3_), 3.56–3.91 (m, 12H, OCH_3_, CH_2,_), 4.11 (m, 0.35 × 2H, OCH_2_), 4.27 (m, 0.30 × 2H, OCH_2_), 4.47 (d, 0.35 × 2H, *J* = 8.0 Hz, OCH_2_), 6.43–7.20 (m, 20H, ArH), OH not observed. ^13^C-NMR (100 MHz, CDCl_3_): δ 13.19, 13.23, 20.83, 24.45, 25.12, 25.22, 33.43, 33.60, 38.59, 48.65, 55.47, 55.60, 60.50, 105.49, 112.62, 113.37, 113.98, 114.68, 125.45, 127.37, 127.39, 128.42, 128.44, 128.91, 129.26, 130.14, 130.25, 131.53, 131.62, 131.87, 135.79, 137.43, 142.25, 152.79, 153.99, 154.93, 156.78. IR: ν_max_ (KBr) cm^−1^: 3425.1, 3327.1, 2929.2, 2850.7, 1626.1, 1579.8, 1507.5, 1449.4, 1411.5, 1310.5, 1242.3, 1170.2, 1127.5, 892.5, 834.3, 641.0. HRMS (EI): Found 706.3118 (M + Na)^+^, C_44_H_45_NO_6_Na requires 706.3145.

#### 2.1.28. (*E*)-*N*-(2-{4-[(*E/Z*)-1,2-Bis-(4-hydroxyphenyl)but-1-enyl]phenoxy}ethyl)-*N*-methyl-3-p-tolyl-2-(3,4,5-trimethoxyphenyl)acrylamide **38**

The acrylic acid analogue **19** was reacted with the endoxifen derivative **10**, following the general method above. The product **38** was afforded as a brown oil (99 mg, 92%), then changes to a semi-solid resin. ^1^H-NMR (400 MHz, CDCl_3_): δ 0.90–0.93 (m, 3H, CH_3_), 2.32 (s, 3H, CH_3_), 2.44–2.46 (m, 2H, CH_2_), 3.08–3.23 (m, 3H, NCH_3_), 3.49–3.92 (m, 12H, OCH_3_, CH_2,_), 4.08 (m, 0.31 × 2H, OCH_2_), 4.25 (m, 0.27 × 2H, OCH_2_), 4.51 (d, 0.42 × 2H, *J =* 8.0 Hz, OCH_2_), 6.42–7.14 (m, 19H, ArH), 2xOH not observed. ^13^C-NMR (100 MHz, CDCl_3_): δ 13.28, 20.83, 24.44, 25.11, 33.42, 48.67, 55.46, 55.68, 60.55, 105.32, 105.47, 112.61, 113.37, 113.99, 114.49, 114.53, 114.65, 128.44, 128.73, 128.88, 130.15, 130.29, 130.48, 131.52, 131.59, 133.59, 134.94, 137.49, 140.06, 152.72, 152.81, 153.12, 153.74, 154.17, 154.66, 156.88. IR: ν_max_ (KBr) cm^−1^: 3428.8, 3328.5, 2929.5, 2850.8, 1625.9, 1580.6, 1509.4, 1449.8, 1411.3, 1310.5, 1241.6, 1169.9, 1127.4, 832.9. HRMS (EI): Found 722.3099 (M + Na)^+^, C_44_H_45_NO_7_Na requires 722.3094.

#### 2.1.29. (*E*)-*N*-(2-{4-[(*E/Z*)-1-(4-Hydroxyphenyl)-2-phenylbut-1-enyl]phenoxy}ethyl)-*N*-methyl-3-(4-methylsulfanylphenyl)-2-(3,4,5-trimethoxyphenyl) acrylamide **39**

The acrylic acid analogue **20** was reacted with the endoxifen derivative **9**, following the general method above. The product **39** was afforded as a brown oil (99 mg, 90%), then changes to a semi-solid resin. ^1^H-NMR (400 MHz, CDCl_3_): δ 0.91–0.93 (m, 3H, CH_3_), 2.45–2.50 (m, 5H, CH_2_), 3.07–3.22 (m, 3H, NCH_3_), 3.57–3.89 (m, 11H, OCH_3_, CH_2_), 4.10 (m, 0.33 × 2H, OCH_2_), 4.26 (m, 0.35 × 2H, OCH_2_), 4.48 (d, 0.32 × 2H, *J =* 8.0 Hz, OCH_2_), 6.50–7.18 (m, 20H, ArH), OH not observed. ^13^C-NMR (100 MHz, CDCl_3_): δ 13.20, 13.24, 14.95, 24..45, 25.13, 25.23, 33.43, 48.64, 55.53, 55.82, 60.51, 105.40, 112.64, 113.38, 113.98, 114.69, 125.21, 125.45, 127.37, 129.24, 129.39, 130.13, 130.25, 131.52, 131.62, 136.16, 137.38, 138.18, 138.24, 152.92, 154.02, 154.95, 156.79. IR: ν_max_ (KBr) cm^−1^: 3326.9, 2929.2, 2850.8, 1626.1, 1579.2, 1507.6, 1449.4, 1409.1, 1312.0, 1242.1, 1170.1, 1127.3, 1050.0, 1005.7, 892.5, 834.0, 701.8. HRMS (EI): Found 738.2897 (M + Na)^+^, C_44_H_45_NO_6_NaS requires 738.2865.

#### 2.1.30. (*E*)-*N*-(2-{4-[(*E/Z*)-1,2-Bis-(4-hydroxyphenyl)but-1-enyl]phenoxy}ethyl)-*N*-methyl-3-(4-methylsulfanylphenyl)-2-(3,4,5-trimethoxyphenyl)acrylamide **40**

The acrylic acid analogue **20** was reacted with the endoxifen derivative **10**, following the general method above. The product **40** was afforded as a brown oil (103 mg, 91%), then changes to a semi-solid resin. ^1^H-NMR (400 MHz, CDCl_3_): δ 0.90–0.93 (m, 3H, CH_3_), 2.42–2.45 (m, 5H, CH_2_), 3.07–3.22 (m, 3H, NCH_3_), 3.52–3.89 (m, 11H, OCH_3_, CH_2,_), 4.08 (m, 0.27 × 2H, OCH_2_), 4.25 (m, 0.28 × 2H, OCH_2_), 4.50 (d, 0.45 × 2H, *J =* 8.0 Hz, OCH_2_), 6.48–7.11 (m, 19H, ArH), 2xOH not observed. ^13^C-NMR (100 MHz, CDCl_3_): δ 13.28, 14.91, 24..43, 25.10, 33.40, 48.68, 55.52, 60.56, 105.23, 105.37, 112.61, 113.37, 113.99, 114.52, 114.65, 125.20, 129.41, 130.15, 130.30, 131.53, 133.58, 152.92, 153.74, 154.09, 154.17, 154.65, 156.89. IR: ν_max_ (KBr) cm^−1^: 3376.8, 3327.7, 2929.4, 2850.9, 1626.0, 1607.1, 1580.3, 1509.0, 1449.7, 1409.1, 1312.3, 1241.6, 1170.0, 1127.3, 892.8, 833.2. HRMS (EI): Found 754.2819 (M + Na)^+^, C_44_H_45_NO_7_NaS requires 754.2814.

#### 2.1.31. (*E*)-3-(4-Bromophenyl)-*N*-(2-{4-[(*E/Z*)-1-(4-hydroxyphenyl)-2-phenylbut-1-enyl]phenoxy}ethyl)-*N*-methyl-2-(3,4,5-trimethoxyphenyl)acrylamide **41**

The acrylic acid analogue **17** was reacted with the endoxifen derivative **9**, following the general method above. The product **41** was afforded as a brown oil (105 mg, 91%), then changes to a semi-solid resin. ^1^H-NMR (400 MHz, CDCl_3_): δ 0.91–0.94 (m, 3H, CH_3_), 2.47–2.50 (m, 2H, CH_2_), 3.05–3.21 (m, 3H, NCH_3_), 3.56–4.47 (m, 13H, OCH_3_, CH_2_), 6.42–7.35 (m, 20H, ArH), OH not observed. ^13^C-NMR (100 MHz, CDCl_3_): δ 13.18, 13.26, 24.44, 25.12, 25.22, 33.43, 48.66, 55.52, 60.52, 105.35, 112.63, 113.38, 113.98, 114.68, 121.42, 121.44, 125.46, 127.38, 129.24, 129.26, 130.13, 130.27, 130.53, 130.89, 130.91, 131.52, 131.62, 133.81, 137.35, 137.59, 140.37, 142.10, 142.25, 152.96, 154.02, 154.94, 156.78, 162.19. IR: ν_max_ (KBr) cm^−1^: 3327.0, 2929.3, 2850.8, 1626.2, 1579.0, 1507.5, 1449.4, 1409.0, 1311.9, 1242.4, 1170.5, 1127.9, 1009.7, 892.5, 834.3. HRMS (EI): Found 770.2120 (M + Na)^+^, C_43_H_42_BrNO_6_Na requires 770.2094.

#### 2.1.32. (*E*)-*N*-(2-{4-[(*E/Z*)-1,2-Bis-(4-hydroxyphenyl)but-1-enyl]phenoxy}ethyl)-3-(4-bromophenyl)-*N*-methyl-2-(3,4,5-trimethoxyphenyl)acrylamide **42**

The acrylic acid analogue **17** was reacted with the endoxifen derivative **10**, following the general method above. The product **42** was afforded as a brown oil (106 mg, 90%), then changes to a semi-solid resin. ^1^H-NMR (400 MHz, CDCl_3_): δ 0.90–0.93 (m, 3H, CH_3_), 2.38–2.48 (m, 2H, CH_2_), 3.08–3.20 (m, 3H, NCH_3_), 3.51–4.51 (m, 13H, OCH_3_, CH_2,_), 6.41–7.35 (m, 19H, ArH), 2xOH not observed. ^13^C-NMR (100 MHz, CDCl_3_): δ 13.28, 24.43, 25.10, 33.41, 48.70, 55.46, 55.51, 55.62, 60.57, 105.19, 105.32, 112.59, 113.36, 114.00, 114.47, 114.52, 114.66, 121.48, 129.41, 129.79, 129.95, 130.15, 130.30, 130.53, 130.91, 131.52, 131.60, 133.59, 133.73, 135.29, 136.55, 137.15, 137.49, 152.88, 152.96, 153.02, 153.73, 154.08, 154.15, 154.64, 156.89, 171.26. IR: ν_max_ (KBr) cm^−1^: 3327.2, 2929.2, 2850.8, 1626.4, 1508.9, 1508.9, 1449.3, 1409.2, 1311.9, 1242.9, 1169.9, 1127.9, 1009.9, 892.5, 833.9, 641.2. HRMS (EI): Found 786.2071 (M + Na)^+^, C_43_H_42_BrNO_7_Na requires 786.2043.

#### 2.1.33. (*E*)-3-(3-Fluoro-4-methoxyphenyl)-*N*-(2-{4-[(*E/Z*)-1-(4-hydroxyphenyl)-2-phenylbut-1-enyl]phenoxy}ethyl)-*N*-methyl-2-(3,4,5-trimethoxyphenyl) acrylamide **43**

The acrylic acid analogue **15** was reacted with the endoxifen derivative **9**, following the general method above. The product **43** was afforded as a brown oil (103 mg, 93%), then changes to a semi-solid resin. ^1^H-NMR (400 MHz, CDCl_3_): δ 0.91–0.93 (m, 3H, CH_3_), 2.47–2.50 (m, 2H, CH_2_), 3.06–3.21 (m, 3H, NCH_3_), 3.60–3.86 (m, 14H, OCH_3_, CH_2,_), 4.10 (m, 0.34 × 2H, OCH_2_), 4.25 (m, 0.27 × 2H, OCH_2_), 4.43 (d, 0.35 × 2H, *J =* 8.0 Hz, OCH_2_), 6.43–7.19 (m, 19H, ArH), OH not observed. ^13^C-NMR (100 MHz, CDCl_3_): δ 13.17, 13.21, 24.44, 25.11, 28.55, 28.63, 33.42, 48.66, 53.02, 55.58, 55.69, 60.53, 105.41, 112.26, 112.59, 113.37, 113.95, 114.66, 116.19, 116.38, 125.45, 125.55, 127.36, 129.23, 130.13, 130.24, 131.52, 131.60, 134.58, 135.94, 137.29, 140.39, 142.10, 146.82, 146.93, 150.04, 152.46, 152.97, 153.94, 154.88, 156.75, 171.31. IR: ν_max_ (KBr) cm^−1^: 3327.6, 2929.2, 2850.8, 1626.4, 1578.4, 1509.0, 1437.1, 1311.2, 1272.5, 1242.7, 1127.2, 892.8, 834.9. HRMS (EI): Found 740.2983 (M + Na)^+^, C_44_H_44_NO_7_NaF requires 740.3000.

#### 2.1.34. (*E*)-*N*-(2-{4-[(*E/Z*)-1,2-Bis-(4-hydroxyphenyl)but-1-enyl]phenoxy}ethyl)-3-(3-fluoro-4-methoxyphenyl)-*N*-methyl-2-(3,4,5-trimethoxyphenyl)acrylamide **44**

The acrylic acid analogue **15** was reacted with the endoxifen derivative **10**, following the general method above. The product **44** was afforded as a brown oil (99 mg, 88%), then changes to a semi-solid resin. ^1^H-NMR (400 MHz, CDCl_3_): δ 0.90–0.93 (m, 3H, CH_3_), 2.41–2.45 (m, 2H, CH_2_), 3.07–3.21 (m, 3H, NCH_3_), 3.48–4.48 (m, 16H, OCH_3_, CH_2,_), 6.44–7.14 (m, 19H, ArH), 2xOH not observed. ^13^C-NMR (100 MHz, CDCl_3_): δ 13.26, 24.43, 25.10, 33.41, 48.69, 55.58, 55.71, 60.56, 60.59, 105.24, 105.38, 112.29, 112.60, 113.36, 113.98, 114.51, 114.65, 116.21, 116.34, 125.59, 127.66, 129.70, 130.15, 130.26, 131.52, 133.61, 146.87, 146.98, 150.03, 152.93, 153.02, 153.71, 154.07, 154.14, 154.63, 156.85. IR: ν_max_ (KBr) cm^−1^: 3376.8, 3328.3, 2930.5, 2850.8, 1608.1, 1581.4, 1511.1, 1463.1, 1410.9, 1302.2, 1274.2, 1239.5, 1169.9, 1126.8, 1050.7, 1025.9, 902.0, 832.9. HRMS (EI): Found 756.2915 (M + Na)^+^, C_44_H_44_NO_8_NaF requires 756.2949.

#### 2.1.35. (*E*)-3-(3-Amino-4-methoxyphenyl)-*N*-(2-{4-[(*E/Z*)-1-(4-hydroxyphenyl)-2-phenylbut-1-enyl]phenoxy}ethyl)-*N*-methyl-2-(3,4,5-trimethoxyphenyl) acrylamide **45**

The acrylic acid analogue **16** was reacted with the endoxifen derivative **9**, following the general method above. The product **45** was afforded as a brown oil (100 mg, 91%), then changes to a semi-solid resin. ^1^H-NMR (400 MHz, CDCl_3_): δ 0.91–0.95 (m, 3H, CH_3_), 2.47–2.50 (m, 2H, CH_2_), 3.06–3.22 (m, 3H, NCH_3_), 3.51–3.88 (m, 16H, OCH_3_, CH_2,_, NH_2_), 4.10 (m, 0.32 × 2H, OCH_2_), 4.26 (m, 0.29 × 2H, OCH_2_), 4.51 (d, 0.39 × 2H, *J =* 8.0 Hz, OCH_2_), 6.39–7.18 (m, 19H, ArH), OH not observed. ^13^C-NMR (100 MHz, CDCl_3_): δ 13.19, 13.23, 24.46, 25.13, 25.21, 28.64, 33.44, 48.65, 55.01, 55.57, 60.50, 105.53, 109.39, 112.64, 113.40, 113.97, 114.67, 115.18, 120.17, 125.44, 127.37, 129.09, 129.26, 130.14, 130.24, 131.53, 131.60, 135.09, 142.14, 142.24, 146.81, 152.82, 153.95, 154.90, 156.67. IR: ν_max_ (KBr) cm^−1^: 3327.2, 2929.4, 2850.7, 1625.9, 1579.2, 1508.9, 1449.3, 1411.2, 1310.6, 1242.1, 1169.5, 1126.1, 1046.3, 892.7, 833.9. HRMS (EI): Found 737.3238 (M + Na)^+^, C_44_H_46_N_2_O_7_Na requires 737.3203.

#### 2.1.36. (*E*)-3-(3-Amino-4-methoxyphenyl)-*N*-(2-{4-[(*E/Z*)-1,2-bis-(4-hydroxyphenyl)but-1-enyl]phenoxy}ethyl)-*N*-methyl-2-(3,4,5-trimethoxyphenyl) acrylamide **46**

The acrylic acid analogue **16** was reacted with the endoxifen derivative **10**, following the general method above. The product **46** was afforded as a brown oil (106 mg, 94%), then changes to a semi-solid resin. ^1^H-NMR (400 MHz, CDCl_3_): δ 0.91–0.94 (m, 3H, CH_3_), 2.44–2.46 (m, 2H, CH_2_), 3.08–3.22 (m, 3H, NCH_3_), 3.51–4.34 (m, 18H, OCH_3_, CH_2_, NH_2_), 6.49–7.12 (m, 18H, ArH), 2xOH not observed. ^13^C-NMR (100 MHz, CDCl_3_): δ 13.27, 24.46, 25.13, 29.26, 33.44, 48.70, 55.03, 55.26, 55.56, 55.82, 60.55, 105.42, 106.23, 109.40, 113.39, 113.93, 114.36, 114.44, 114.51, 114.61, 130.17, 130.24, 130.31, 131.53, 133.62, 152.82, 153.85, 154.57, 156.61. IR: ν_max_ (KBr) cm^−1^: 3327.3, 2929.2, 2850.7, 1625.8, 1578.6, 1510.6, 1436.5, 1311.1, 1242.9, 1169.1, 1126.2, 892.6, 833.8. HRMS (EI): Found 753.3172 (M + Na)^+^, C_44_H_46_N_2_O_8_Na requires 753.3152.

#### 2.1.37. (*E*)-2-(4-Methoxyphenyl)-1-(piperidin-1-yl)-3-(3,4,5-trimethoxyphenyl)prop-2-en-1-one **47**

To a suspension of **23** (1.45 mmol, 0.5 g) in anhydrous dichloromethane (20 mL), 2-chloro-1-methylpyridinium iodide (4.35 mmol 1.11 g) was added and the reaction mixture was stirred for 5 min at room temperature. To this was added piperidine (1.45 mmol, 0.12 g, 0.14 mL) followed by the addition of triethylamine (7.25 mmol, 0.73 g, 1.01 mL). The reaction mixture was stirred at room temperature for 1 h and was then diluted with 10% hydrochloric acid (10 mL), washed with 10% sodium hydroxide (10 mL), water and brine and dried over sodium sulphate. The crude product was purified by column chromatography (dichloromethane:ethyl acetate, 1:1) to afford the product as a yellow powder, 48% (0.287 g), Mp 106–108 °C, HPLC 95%. IR: ν_max_ (KBr) cm^−1^: 2993, 2948, 2865, 2835, 1615, 1599, 1577, 1505, 1453, 1432, 1329, 1281, 1238, 1125, 1021, 989, 904, 845, 833, 718, 657. ^1^H NMR (400 MHz, CDCl_3_) δ 1.32 (br. s., 2 H, CH_2_), 1.57 (br. s., 4 H, CH_2_), 3.46 (br. s., 2 H, CH_2_), 3.54 (d, *J* = 2.49 Hz, 2 H, CH_2_), 3.58 (s, 6 H, CH_3_), 3.76 (s, 3 H, CH_3_), 3.78 (s, 3 H, CH_3_), 6.35 (s, 2 H, Ar-H), 6.50 (s, 1 H, CH), 6.81 (d, *J* = 8.71 Hz, 2 H, Ar-H), 7.27 (d, *J* = 8.71 Hz, 2 H, Ar-H). ^13^C NMR (101 MHz, CDCl_3_) δ 24.51 (3 × CH_2_), 55.24 (2 × CH_2_), 55.73 (3 × CH_3_), 60.82 (CH_3_), 106.55 (2 × CH), 114.00 (2 × CH), 127.99 (C), 128.29 (C), 130.21 (2 × CH), 131.02 (C), 137.00 (CH), 137.47 (C), 152.66 (2C), 159.22 (C), 170.09 (C=O). HRMS (EI): Found 434.1929 (M + Na)^+^, C_24_H_29_NO_5_ requires 434.1944.

#### 2.1.38. (*E*)-2-(4-methoxyphenyl)-1-(pyrrolidin-1-yl)-3-(3,4,5-trimethoxyphenyl)prop-2-en-1-one **48**

The acrylic acid **23** (1.45 mmol 0.5 g) was reacted with 2-chloro-1-methylpyridinium iodide (4.35 mmol 1.11 g), pyrrolidine (1.45 mmol, 0.1 g, 0.12 mL) and triethylamine (7.25 mmol, 0.73 g, 1.01 mL) in anhydrous dichloromethane (20 mL) as described for compound **47** above. The crude product was purified by column chromatography (dichloromethane: ethyl acetate,1:1) to afford the product as a yellow oil, 44% (0.256 g); HPLC; 91%. IR: ν_max_ (KBr) cm^−1^: 3463, 2937, 2877, 2837, 1605, 1578, 1505, 1450, 1416, 1327, 1239, 1121, 1005, 913, 872, 836, 770. ^1^H NMR (400 MHz, CDCl_3_) δ 1.80–1.89 (m, 4 H, CH_2_), 3.26 (t, *J* = 6.22 Hz, 2 H, CH_2_), 3.55 (t, *J* = 6.43 Hz, 2 H, CH_2_), 3.61 (s, 6 H, CH_3_), 3.80 (s, 3 H, CH_3_), 3.82 (s, 3 H, CH_3_), 6.37 (s, 2 H, Ar-H), 6.74 (s, 1 H, CH), 6.83–6.88 (m, 2 H, Ar-H), 7.27–7.30 (m, 2 H, Ar-H). ^13^C NMR (101 MHz, CDCl_3_) δ 24.27 (CH_2_), 26.13 (CH_2_), 46.03 (CH_2_), 48.16 (CH_2_), 55.27 (CH_3_), 55.72 (2 × CH_3_), 60.83 (CH_3_), 106.73 (2 × CH), 114.04 (2 × CH), 127.86 (C), 130.34 (2 × CH), 130.63 (C), 137.58 (CH), 137.88 (C), 152.63 (2 × C), 159.21 (C), 170.03 (C=O). HRMS (EI): Found 420.1794 (M + Na)^+^, C_23_H_27_NO_5_ requires 420.1787.

#### 2.1.39. (*E*)-3-(3-hydroxy-4-methoxyphenyl)-1-(piperidin-1-yl)-2-(3,4,5-trimethoxyphenyl)prop-2-en-1-one **49**

The acrylic acid **13** (1.45 mmol 0.5 g) was reacted with 2-chloro-1-methylpyridinium iodide (4.35 mmol 1.11 g), piperidine (1.38 mmol, 0.12 g, 0.13 mL) and triethylamine (7.25 mmol, 0.73 g, 1.01 mL) in anhydrous dichloromethane (20 mL) as described for compound **47** above. The crude product was purified by column chromatography (dichloromethane: ethyl acetate,1:1) to afford the product as a yellow oil, 6.5% (0.038 g), HPLC 92%. IR: ν_max_ (KBr) cm^−1^: 3209, 2998, 2936, 2854, 2250, 1605, 1578, 1505, 1440, 1256, 1234, 1120, 1024, 999, 907, 852, 803, 772, 725. ^1^H NMR (400 MHz, CDCl_3_) δ 1.32 (br. s., 2 H, CH_2_), 1.55 (br. s., 4 H, CH_2_), 3.43 (br. s., 2 H, CH_2_),3.54 (br. s., 2 H, CH_2_), 3.65 (s, 6 H, CH_3_), 3.78 (s, 3 H, CH_3_), 3.79 (s, 3 H, CH_3_), 6.46 (s, 1 H, CH), 6.51 (s, 2 H, Ar-H), 6.60 (d, *J* = 0.83 Hz, 2 H, Ar-H), 6.72 (s, 1 H,Ar-H). ^13^C NMR (101 MHz, CDCl_3_) δ 24.55 (3 × CH_2_), 55.88 (C), 56.14 (CH_3_), 60.93 (2 × CH_3_), 106.09 (2 × CH), 110.16 (CH), 115.42 (CH), 121.82 (CH), 128.77 (2 × C), 131.03 (C), 136.11 (CH), 137.82 (C), 145.10 (2 × C), 153.23 (2 × C), 170.09 (C=O). HRMS (EI): Found 450.1898 (M + Na)^+^, C_24_H_29_NO_6_ requires 450.1893.

#### 2.1.40. (*E*)-3-(4-Methoxy-3-nitrophenyl)-1-(piperidin-1-yl)-2-(3,4,5-trimethoxyphenyl)prop-2-en-1-one **50**

The acrylic acid **21** (1.45 mmol 0.5 g) was reacted with 2-chloro-1-methylpyridinium iodide (4.35 mmol 1.11 g), piperidine (1.38 mmol, 0.12 g, 0.13 mL) and triethylamine (7.25 mmol, 0.73 g, 1.01 mL) in anhydrous dichloromethane (20 mL) as described for compound **47** above. The crude product was purified by column chromatography (ethyl acetate:methanol, 9:1) to afford the product as a yellow oil, 31% (0.177 g), HPLC 67%. IR: ν_max_ (KBr) cm^−1^: 2937, 2854, 1614, 1578, 1528, 1503, 1440, 1411, 1279, 1234, 1122, 999, 915, 827, 732, 675. ^1^H NMR (400 MHz, CDCl_3_) δ 1.40 (br. s., 2 H, CH_2_), 1.63 (br. s., 4 H, CH_2_), 3.48 (br. s., 2 H, CH_2_), 3.63 (br. s., 2 H, CH_2_), 3.73 (s, 6 H, CH_3_), 3.88 (s, 3 H, CH_3_), 3.93 (s, 3 H, CH_3_), 6.55 (s, 1 H, CH), 6.56 (s, 2 H, Ar-H), 6.89 (d, *J* = 8.71 Hz, 1 H, Ar-H), 7.29 (d, *J* = 2.49 Hz, 1 H, Ar-H), 7.70 (d, *J* = 2.07 Hz, 1 H, Ar-H). ^13^C NMR (101 MHz, CDCl_3_) δ 24.47 (3 × CH_2_), 56.25 (2 × CH_2_), 56.57 (3 × CH_3_), 61.02 (CH_3_), 105.78 (2 × CH), 113.05 (CH), 126.00 (CH), 126.53 (C), 128.07 (C), 130.13 (C), 134.91 (CH), 138.35 (CH), 138.92 (C), 152.13 (C), 153.64 (2 × C), 169.16 (C=O). HRMS (EI): Found 457.1974 (M + H)^+^, C_24_H_28_N_2_O_7_ requires 457.1975.

#### 2.1.41. (*E*)-3-(4-Methoxy-3-nitrophenyl)-1-(pyrrolidin-1-yl)-2-(3,4,5-trimethoxyphenyl)prop-2-en-1-one **51**

The acrylic acid **21** (1.45 mmol 0.5 g) was reacted with 2-chloro-1-methylpyridinium iodide (4.35 mmol 1.11 g), pyrrolidine (1.38 mmol, 0.12 g, 0.13 mL) and triethylamine (7.25 mmol, 0.73 g, 1.01 mL) in anhydrous dichloromethane (20 mL) as described for compound **47** above. The crude product was purified by column chromatography (dichloromethane:ethyl acetate, 1:1) to afford the product as a yellow oil, 52% (0.292 g). HPLC 98% and was used in the following reaction without further purification. IR: ν_max_ (KBr) cm^−1^: 3463, 2971, 2942, 2878, 2841, 1613, 1578, 1528, 1503, 1410, 1353, 1236, 1122, 1087, 1005, 912, 829, 808, 751, 670. ^1^H NMR (400 MHz, CDCl_3_) δ 1.85–1.92 (m, 4 H, CH_2_), 3.30 (t, *J* = 6.22 Hz, 2 H, CH_2_), 3.57 (t, *J* = 6.43 Hz, 2 H, CH_2_), 3.74 (s, 6 H, CH_3_), 3.88 (s, 3 H, CH_3_), 3.93 (s, 3 H, CH_3_), 6.55 (s, 2 H, Ar-H), 6.73 (s, 1 H, CH), 6.89 (d, *J* = 9.12 Hz, 1 H, Ar-H), 7.25 (d, *J* = 2.07 Hz, 1 H, Ar-H), 7.68 (d, *J* = 2.07 Hz, 1 H, Ar-H). ^13^C NMR (101 MHz, CDCl_3_) δ 24.26 (2 × CH_2_), 26.13 (CH_2_), 46.12 (CH_2_), 48.11 (CH_2_), 56.29 (2 × CH_3_), 56.57 (CH_3_), 61.04 (CH_3_), 106.11 (2 × CH), 113.03 (CH), 126.62 (CH), 127.79 (C), 128.07 (C), 129.96 (C), 135.07 (CH), 138.34 (CH),139.77 (C), 152.22 (C), 153.70 (2 × C), 169.01 (C=O).

#### 2.1.42. (*E*)-3-(4-Methoxyphenyl)-1-(pyrrolidin-1-yl)-2-(3,4,5-trimethoxyphenyl)prop-2-en-1-one **52**

The acrylic acid **14** (1.45 mmol 0.5 g) was reacted with 2-chloro-1-methylpyridinium iodide (4.35 mmol 1.11 g), pyrrolidine (1.38 mmol, 0.12 g, 0.13 mL) and triethylamine (7.25 mmol, 0.73 g, 1.01 mL) in anhydrous dichloromethane (20 mL) as described for compound **47** above. The crude product was purified by column chromatography (dichloromethane:ethyl acetate, 1:1) to afford the product as a clear oil 37% (0.215 g), HPLC 96%. IR: ν_max_ (KBr) cm^−1^: 3463, 2936, 2873, 2837, 1603, 1577, 1505, 1431, 1235, 1175, 1122, 1027, 1004, 828, 665. ^1^H NMR (400 MHz, CDCl_3_) δ 1.82–1.92 (m, 4 H, CH_2_), 3.31 (t, *J* = 6.22 Hz, 2 H, CH_2_), 3.56 (t, *J* = 6.43 Hz, 2 H, CH_2_), 3.71 (s, 6 H, CH_3_), 3.77 (s, 3 H, CH_3_), 3.87 (s, 3 H, CH_3_), 6.57 (s, 2 H, Ar-H), 6.70–6.75 (m, 2 H, Ar-H), 6.77 (s, 1 H, CH), 7.08 (m, *J* = 8.71 Hz, 2 H, Ar-H). ^13^C NMR (101 MHz, CDCl_3_) δ 24.30 (CH_2_), 26.14 (CH_2_), 46.02 (CH_2_), 48.17 (CH_2_), 55.19 (CH_3_), 56.12 (2 × CH_3_), 60.94 (CH_3_), 106.32 (2 × CH), 113.46 (2 × CH), 127.86 (C), 130.60 (C), 130.94 (2 × CH), 136.69 (CH) 137.65 (C), 153.31 (2C), 159.21 (C), 169.97 (C=O). HRMS (EI): Found 398.1974 (M + H), C_23_H_28_NO_5_ requires 398.1967.

#### 2.1.43. (*E*)-3-(4-Methoxyphenyl)-1-(piperidin-1-yl)-2-(3,4,5-trimethoxyphenyl)prop-2-en-1-one **53**

The acrylic acid **14** (1.45 mmol 0.5 g) was reacted with 2-chloro-1-methylpyridinium iodide (4.35 mmol 1.11 g), piperidine (1.38 mmol, 0.12 g, 0.13 mL) and triethylamine (7.25 mmol, 0.73 g, 1.01 mL) in anhydrous dichloromethane (20 mL) as described for compound **47** above. The reaction mixture was stirred at room temperature for 1 h and was then diluted with 10% hydrochloric acid (10 mL), washed with 10% sodium hydroxide (10 mL), water and brine and dried over sodium sulphate. The crude product was purified by column chromatography (dichloromethane: ethyl acetate,1:1) to afford the product as a pale yellow oil, 38% (0.225 g), HPLC 99%. IR: ν_max_ (KBr) cm^−1^: 3490, 2996, 2854, 2838, 1619, 1605, 1577, 1506, 1433, 1410, 1234, 1122, 1004, 996, 827, 716, 704, 554. ^1^H NMR (400 MHz, CDCl_3_) δ 1.40 (br. s., 2 H, CH_2_), 1.64 (br. s., 4 H, CH_2_), 3.53 (br. s., 2 H, CH_2_), 3.56–3.68 (m, 2 H, CH_2_), 3.71 (s, 6 H, CH_3_), 3.77 (s, 3 H, CH_3_), 3.87 (s, 3 H, CH_3_), 6.57 (s, 2 H, Ar-H), 6.59 (s, 1 H, CH), 6.71–6.77 (m, 2 H, Ar-H), 7.11 (m, *J* = 8.71 Hz, 2 H,Ar-H). ^13^C NMR (101 MHz, CDCl_3_) δ 24.57 (3 × CH_2_), 55.22 (2 × CH_2_), 56.11 (3 × CH_3_), 60.94 (CH_3_), 105.99 (2 × CH), 113.51 (2 × CH), 127.88 (C), 128.82 (C), 130.80 (2 × CH), 131.25 (C), 135.69 (CH), 137.70 (C), 153.29 (2 × C), 159.15 (C), 170.16 (C=O). HRMS (EI): Found 412.2121 (M + H), C_24_H_29_NO_5_ requires 412.2124.

#### 2.1.44. (*E*)-3-(3-Amino-4-methoxyphenyl)-1-(pyrrolidin-1-yl)-2-(3,4,5-trimethoxyphenyl)prop-2-en-1-one **54**

The acrylic acid **51** (0.34 mmol, 0.150 g) was dissolved in a mixture of ethanol (5 mL) acetic acid (5 mL) and water (2 mL). Hydrochloric acid(1 drop) was added followed by iron powder (3.4 mmol, 0.19 g). The mixture was vigorously stirred under reflux for 12 h. After completion of the reaction, the mixture was filtered through Celite, diluted with NaHCO_3_ solution and extracted with dichloromethane. The organic layers were combined, washed with water and brine and dried over sodium sulphate. The crude product was purified by column chromatography (dichloromethane:ethyl acetate, 1:1) to afford the product as a yellow oil, 79% (0.111 g), HPLC 70%. IR: ν_max_ (KBr) cm^−1^: 3464, 3360, 2938, 2877, 2836, 1578, 1504, 1410, 1230, 1121, 1003, 911, 797, 743, 705. ^1^H NMR (400 MHz, DMSO-*d*_6_) δ 1.74–1.80 (m, 4 H, CH 2) 3.33 (br. s., 2 H, CH_2_), 3.60 (s, 6 H, CH_3_), 3.65 (s, 3 H, CH_3_), 3.68 (s, 3 H, CH_3_) 4.59 (br. s., 2 H, NH_2_), 6.32 (dd, *J* = 8.29, 2.07 Hz, 1 H, Ar-H), 6.48 (d, *J* = 2.07 Hz, 1 H, Ar-H), 6.50 (s, 1 H, CH), 6.51 (s, 2 H, Ar-H), 6.63 (d, *J* = 8.29 Hz, 1 H, Ar-H). ^13^C NMR (101 MHz, DMSO-*d*_6_) δ 23.89 (CH_2_), 25.68 (CH_2_), 47.86 (2 × CH_2_), 55.25 (CH_3_), 55.82 (2 × CH_3_), 60.12 (CH_3_), 106.22 (2 × CH), 110.05 (CH), 114.52 (CH), 117.79 (CH), 127.71 (C), 130.23 (C), 131.18 (C), 135.53 (C),137.11 (CH), 137.15 (C), 146.25 (C), 152.76 (2 × C), 168.81 (C=O). HRMS (EI): Found 413.2078 (M + H), C_23_H_28_N_2_O_5_ requires 413.2076.

#### 2.1.45. (*E*)-3-(3-Amino-4-methoxyphenyl)-1-(piperidin-1-yl)-2-(3,4,5-trimethoxyphenyl)prop-2-en-1-one **55**

The acrylic acid **50** (0.33 mmol, 0.150 g) was dissolved in a mixture of ethanol (5 mL), acetic acid (5 mL) and water (2 mL). Hydrochloric acid(1 drop) was added followed by iron powder (3.4 mmol, 0.19 g) following the procedure described above. The crude product was purified by column chromatography (dichloromethane:ethyl acetate, 1:1) to afford the product as a yellow oil, 81%, (0.114 g). IR: ν_max_ (KBr) cm^−1^: 3329, 2933, 1580, 1507, 1411, 1235, 1123, 1023, 773. ^1^H NMR (400 MHz, DMSO-*d*_6_) δ 1.38 (br. s., 6 H, CH_2_), 3.29 (s, 6 H, CH_3_), 3.46 (d, *J* = 4.98 Hz, 4 H, CH_2_), 3.64 (s, 3 H, CH_3_), 3.75 (s, 3 H, CH_3_), 4.61 (br. s., 2 H, NH_2_), 6.32–6.35 (m, 1 H, Ar-H), 6.45 (s, 1 H, CH), 6.49 (s, 2 H, Ar-H), 6.51 (s, 1 H, Ar-H), 6.64 (d, *J* = 8.29 Hz, 1 H, Ar-H). ^13^C NMR (101 MHz, DMSO-*d*_6_) δ 24.05 (3 × CH_2_), 55.67 (2 × CH_2_), 55.77 (3 × CH_3_), 60.10 (CH_3_), 105.71 (2 × CH), 110.09 (CH), 114.33 (CH), 117.59 (CH), 126.98 (C), 127.66 (C), 131.19 (C), 135.46 (C), 137.18 (CH), 137.27 (C), 146.17 (C), 152.92 (2 × C), 168.95 (C=O). HRMS (EI): found 427.2237 (M + H), C_24_H_30_N_2_O_5_ requires 427.2233.

#### 2.1.46. Stability Study for Compounds **27**, **31** and **32**

Analytical high-performance liquid chromatography (HPLC) stability studies were performed using a Symmetry^®^ column (C_18_, 5 µm, 4.6 × 150 mm), a Waters 2487 Dual Wavelength Absorbance detector, a Waters 1525 binary HPLC pump and a Waters 717 plus Autosampler (Waters Corporation, Milford, MA, USA). Samples were detected at wavelength of 254 nm. All samples were analysed using acetonitrile (80%):water (20%) as the mobile phase over 10 min and a flow rate of 1 mL/min. Stock solutions are prepared by dissolving 5mg of compound in 10 mL of mobile phase. Phosphate buffers at the desired pH values (4, 7.4, and 9) were prepared in accordance with the British Pharmacopoeia monograph 2015. 30 µL of stock solution was diluted with 1 mL of appropriate buffer, shaken and injected immediately. Samples were withdrawn and analysed at time intervals of *t* = 0 min, 5 min, 30 min, 60 min, 90 min, 120 min, 24 h and 48 h.

### 2.2. Biochemical Evaluation

#### 2.2.1. MTT Assay for Measurement of Antiproliferative Effects in MCF-7 and MDA-MB-231 Cell Lines

The human breast carcinoma cell line, MCF-7, was purchased from the European Collection of Animal Cell Cultures (ECACC). The cells were maintained in MCF-7 complete medium; consisting of Eagle’s Minimum Essential Medium (MEM) supplemented with 10% (*v*/*v*) Foetal Bovine Serum (FBS), 2 mM l-glutamine, 100 μg/mL penicillin/streptomycin and 1% (*v*/*v*) non-essential amino acids. Cell cultures were maintained at 37 °C under a humidified atmosphere of 5% CO_2_/95% O_2_. The human breast carcinoma cell line, MDA, was purchased from the European Collection of Animal Cell Cultures (ECACC). The cells were maintained in MDA complete medium; consisting of Dulbecco’s Modified Eagle’s Medium (D-MEM) supplemented with 10% (*v*/*v*) Foetal Bovine Serum (FBS), 2 mM l-glutamine, 100 μg/mL penicillin/streptomycin. Cell cultures were maintained at 37 °C under a humidified atmosphere of 5% CO_2_/95% O_2_. The MTT assay was performed according to the reported protocol. The tetrazolium salt, 3-(4,5-dimethylthiazol-2-yl)-2,5-diphenyltetrazolium bromide (MTT) is taken up only by metabolically active cells and cleaved to form a formazan dye by mitochondrial dehydrogenases [30]. Assays where repeated in three experiments performed in triplicate (unless otherwise stated) and reported results represent the mean value ± standard error mean. Graphs of percentage cell viability versus concentration of the subject compound were processed using PRISM [31].

#### 2.2.2. Lactate Dehydrogenase Assay for Measurement of Cytotoxicity

In this assay, the release of cytoplasmic lactate dehydrogenase (LDH) is used as a measure of cell lysis. MCF-7 and MDA-MB-231 cells were seeded at a density of 1 × 10^4^ cells/well in a 96-well plate and incubated for 24 h. The cells were then dosed with 2 μL volumes of the test compounds, over the concentration range 1 nM–50 μM. Cytotoxicity was determined using the CytoTox 96 nonradioactive cytotoxicity assay (Promega, Madison, WI, USA) following the manufacturer’s protocol [32].

#### 2.2.3. Estrogen Receptor Fluorescent Polarisation Assay

Competitive binding affinity experiments were carried out using purified baculovirus-expressed human ERα and ERβ and fluoromone, a fluorescein labelled estrogen ligand. Estrogen receptor binding ability of the selected compounds was investigated using ERα and ERβ fluorescence polarisation-based estrogen receptor competitive assay kits supplied by Invitrogen [33,34]. The assay was performed usibg a protocol described by the manufacturer. The assay allows for high throughput screening of potential ER-subtype ligands. The selected compounds were screened using both the ERα and ERβ competitive assay kits. The protocol for carrying out the assay is similar for both ER subtypes. Principally, the main difference between the kits relates to the functional receptor concentration and the specific activity of the different ERs [33,34].

#### 2.2.4. Ishikawa Cell Line Study

The Ishikawa assay is used to measure estrogen stimulation of alkaline phosphatase enzyme activity (AlkP) by the Ishikawa cell line of human endometrial adenocarcinoma cells. The Ishikawa assay provides a measure of the agonist activity of a compound. The assay was carried out on the lead conjugate, **28**. The assay was carried out following the method of Littlefield et al. [35]. The batch of Ishikawa cells were obtained as a gift from Professor R. Hochberg—who developed the alkaline phosphatase assay in Yale University, CT, USA. Tamoxifen was used as a reference compound in the assay.

#### 2.2.5. NCI One-Dose and Five-Dose Screen Output

The one-dose screen output is reported as a mean graph of the percent growth of treated cells and is similar in appearance to mean graphs generated in the 5-dose assay. The value reported for the one-dose assay is growth relative to the no-drug control and relative to the time zero number of cells. The one-dose assay allows detection of growth inhibition (values between 0 and 100) and lethality (values less than 0). For example, a value of 100 means no growth inhibition. A value of 40 would mean 60% growth inhibition. A value of zero means no net growth over the course of the experiment. A value of −40 would mean 40% lethality. A value of −100 means all cells are dead. The results from the one-dose screen for **28** were manually entered into the COMPARE analysis software via an on-line submission form [36]. The results from the COMPARE analysis are retrievable on-line by searching using the relevant JobID reference number. The COMPARE analysis was run on a database of common anti-cancer agents (JobID: 37472) and the larger more comprehensive database including natural products and other submitted agents (JobID: 37473). Similarly, the results from the five-dose screen for **28** were also manually entered into the COMPARE analysis software via an on-line submission form. The COMPARE analysis was run on a database of common anti-cancer agents (JobID: 37885) and the larger more comprehensive database including natural products and other submitted agents (JobID: 37886).

### 2.3. Molecular Modelling

The lowest energy conformer produced [37] using MACROMODEL [38] was used to generate an ensemble of low energy conformations of **28** in OMEGA [39]. Fifty conformers were generated for the lead conjugate **28** using default parameters and saved as a .pdb file. The resulting .pdb file generated by OMEGA was then utilised by FRED [40] to dock and score the different compound conformers. The protein used to dock the conformers was 3ERT [41] (containing 4-hydroxytamoxifen bound in the human ERα LBD) and 1QKN [42] (containing raloxifene bound in rat ERβ LBD). The active site was defined by a three-dimensional box incorporating the 4-hydroxytamoxifen or raloxifene bound ligand. This box was also extended by five angstroms in each dimension to create additional space to allow for the docking procedure. Each conformer was docked and scored using three functions: Piecewise Linear Potential (PLP), Chemgauss and an updated version, Chemgauss2 [40].

PLP is a heavy atom scoring function; all potentials are based on distances from heavy atom centers (i.e., hydrogen position is irrelevant, although the presence or absence of hydrogen is not, as it can affect the atom typing). PLP recognises atom types such as hydrogen bond donors (i.e., primary and secondary amines), hydrogen bond acceptors (i.e., oxygen and nitrogen atoms with no bound hydrogens), hydroxyl groups (treated as both acceptors and donors) and non-polar entities (i.e., carbon, halogens and nitrogen or sulfur with more than two attached hydrogens). The Chemgauss scoring function combines the Shapegauss scoring function with additional potentials near specific functional groups. The Shapegauss scoring function represents all atoms as smooth Gaussian functions. A pairwise potential between ligand and protein atoms is applied that attempts to maximize their surface contact and minimize their volume overlap. Therefore, the potential is most favourable when the atoms are touching but not overlapping. A correction term is then applied to further penalize atoms that significantly overlap the protein. A consensus of the separate scoring functions is determined and the conformers are ranked accordingly.

The crystal structure of raloxifene, an antagonistic ligand, in rat ERβ isoform (pdb: 1QKN) was used due in this study due to the lack of reported determined co-crystallised antagonist ligands in the human ERβ isoform.

## 3. Results

### 3.1. Synthesis of Endoxifen-Combretastatin Conjugates

Many ER-ligand conjugates reported in the literature contain an agonistic ER-ligand analogue such as estrogen, in their conjugate structure [14,15]. As the goal in our investigation is to develop ER-antagonistic conjugates, possessing minimal agonist activity, only antagonistic ER-ligands were incorporated in the conjugate structural backbone. Endoxifen **11** which together with 4-hydroxytamoxifen is a significant metabolite of tamoxifen, was chosen as a suitable ER-ligand candidate for this study based on its potent ER-binding affinity and antiestrogenic properties [43]; it was also effective in degrading the estrogen receptor [44] (Figure 1). It has also been shown to inhibit aromatase [45] in the MCF-7 human cancer cell line [46]. The structurally related hydroxyendoxifen analogue **12**, previously detected as a metabolite of tamoxifen [47], was also investigated as a potential ER ligand for conjugate design(Figure 1).

**Scheme 1:**
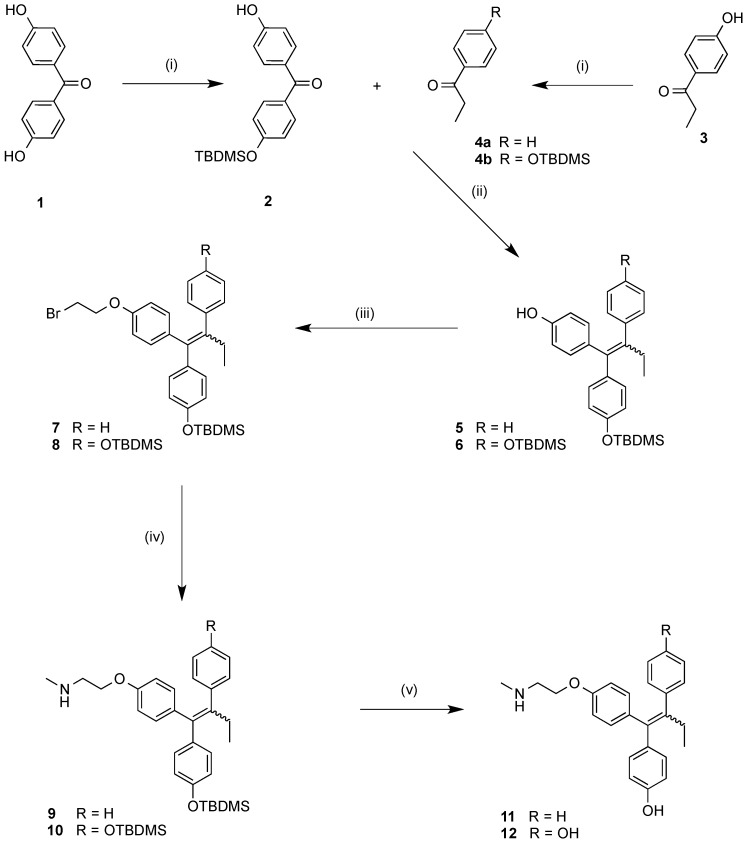
Synthesis of endoxifen **11** and hydroxyendoxifen derivative **12**. Reagents and conditions: (i) TBDMS-Cl, Imidazole, DMF; (ii) Zn, TiCl_4_, THF; (iii) BrCH_2_CH_2_Br, NaOH (aq.), (*n*Bu)_4_NHSO_3_; (iv) CH_3_NH_2_, sealed tube, 60 °C; (v) TBAF, THF. (TBDMS = tert-Butyldimethylsilyl).

A modified multi-step route to the protected endoxifen scaffolds **9** and **10** was developed and is shown in Scheme 1. Initially, the phenolic 4,4′-dihydroxybenzophenone (**1**) was monoprotected as the tert-butyldimethylsilyl-ether (**2**); the 4-hydroxypropiophenone (**3**) was similarly protected to afford (**4b**). The benzophenone (**2**) and propiophenone starting materials (**4a**) and (**4b**) were coupled via a zinc/titanium tetrachloride/tetrahydrofuran McMurray reaction system to afford alkene products **5** and **6** in high yields (93%–98%) containing the triarylethylene ring motif predominant OH directed *E*-isomer [48]. A bromoethylation reaction was carried out using an excess of dibromoethane in the presence of a phase-transfer catalyst((*n*Bu)_4_NHSO_4_) in basic conditions in order to introduce a bromoethylether functionality at the hydroxyl group position on the triarylethylene backbone in moderate yields (52%–54%). Methylamine undergoes reaction with the relevant bromide analogues **7** and **8** in a sealed tube to form the silyl-protected endoxifen **9** [16] and the disilylated endoxifen analogue **10** in moderate to high yields (55%–93%). The silyl ether protecting groups were removed using TBAF to afford the endoxifen **11** and hydroxyendoxifen **12** in high yields (80%–93%). Most of the *E/Z* isomeric mixture ratios of the analogues synthesised were calculated based on the basic side chain OCH_2_ or NCH_2_ signals. Unambiguous assignment of *E/Z* stereochemistry is confirmed using through Nuclear Overhauser Effect (NOE) NMR. In the ^1^H-NMR spectrum of the silyl ether **9** (*Z*:*E* ratio 1:1.3) the *E*-isomer CH_2_N signals are observed as a broad triplet at 2.99 ppm while the *Z*-isomer CH_2_N signals are observed as a triplet further upfield at 2.89 ppm. The *E*-isomer CH_2_O signals are observed as a triplet at 4.11 ppm(*J* = 5 Hz) while the *Z*-isomer CH_2_O signals are observed as a triplet further upfield at 3.95 ppm(*J* = 5 Hz). The relative chemical shifts assigned for the OCH_2_ and NCH_2_ signals for the protons in the basic side chain in the isomeric mixtures are in agreement with reported values for similar compounds [29]. The spectral data confirm that the *trans-*isomer OCH_2_ (and NCH_2_) signals are found further downfield when compared with the *cis-*isomer. Previous studies have demonstrated that 4-hydroxy substituted triarylethylenes such as endoxifen may isomerise under physiological conditions and have little impact on ER binding activity [49,50].

Combretastatin A-4 is an important lead compound in drug development due to its potent antimitotic activity and ability to inhibit the depolymerisation of tubulin(Figure 1) [51]. To date, much work has been carried out in developing combretastatin analogues with potential anticancer applications [52]. The conjugation of combretastatin A4 analogues on steroidal scaffolds and their proapoptotic effects in MCF-7 cells has been recently reported [53]. In this study, a selection of combretastatin acrylic acid derivatives were synthesised using a reported Perkin condensation reaction route [21]*.* The common structural motif amongst the acrylic acid derivatives synthesised was the presence of a carboxylic acid group at either carbon position of the double bond between the two ring systems of the combretastatin core structure. Importantly, the carboxylic acid group allows for further chemical manipulation, such as the formation of amide and ester linkages, which is of interest in our conjugation strategy.

**Scheme 2:**
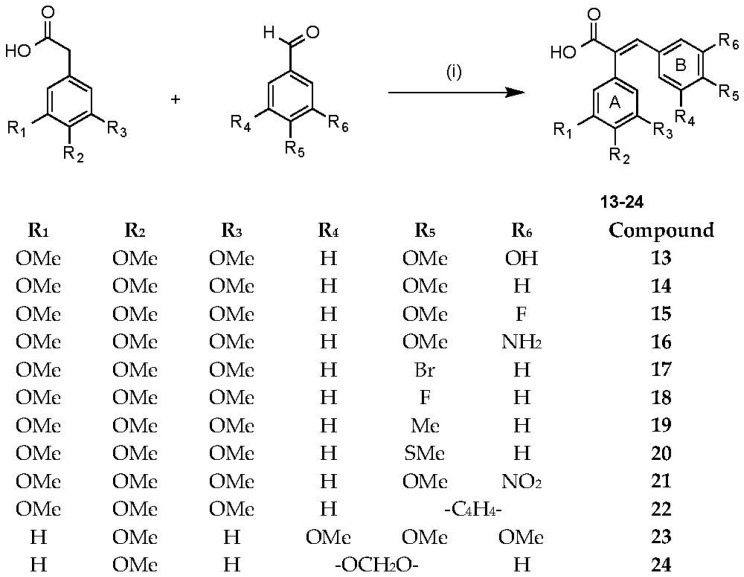
Synthesis of combretastatin acrylic acid analogues. Reagents and conditions: (i) (CH_3_CO)_2_O, Et_3_N, reflux, 3 h followed by conc HCl.

The combretastatin acrylic acids chosen for synthesis were selected based on the biochemical activity available for the “parent” combretastatin analogues [52]—and which differed in structure only by the absence of the acrylic acid’s carboxylic acid functional group [22,54,55,56,57,58,59,60,61,62,63,64,65]. By varying the benzaldehyde and phenylacetic acid in the Perkin condensation reaction, a series of combretastatin acrylic acid analogues **13**–**24** were prepared in yields of 36%–71% (see Scheme 2). In our investigation, combretastatin A-4 was used as a comparison standard for our biochemical evaluation and was synthesised according to a Wittig reaction route reported by Pettit et al. [20].

**Scheme 3:**
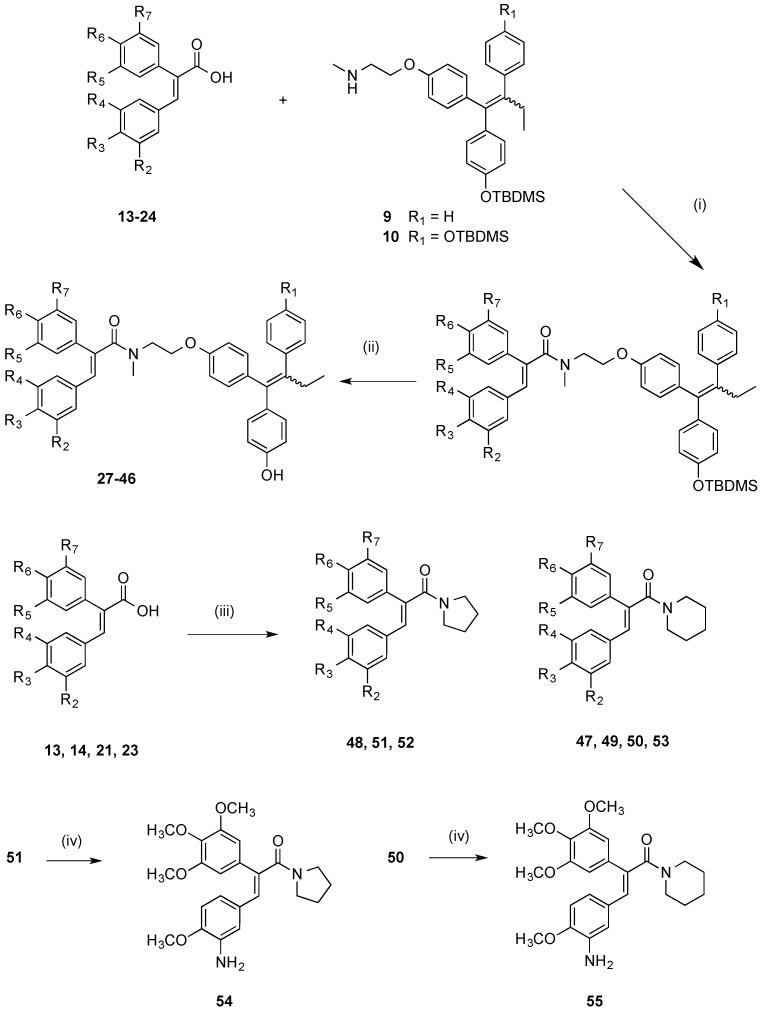
Synthesis of endoxifen-combretastatin hybrid conjugates **27**–**46** and amides **47**–**55**. Reagents and conditions: (i) DCC, HOBt, CH_2_Cl_2_, 20 °C, 24–48 h; (ii) TBAF, THF, 20 °C, 24 h (R_1_–R_8_: see Table 2); (iii) Pyrrolidine or piperidine, 2-chloro-1-methylpyridinium iodide, CH_2_Cl_2_, Et_3_N, 20 °C, 1 h; (iv) Fe, HCl, CH_3_CO_2_H, EtOH, reflux, 12 h.

From the panel of combretastatin type acrylic acids **13**–**24** initially prepared, a subset of the more potent compounds was selected for the synthesis of the direct amide conjugates with endoxifen **11** and hydroxyendoxifen **12**. The amine functional group of the silyl-protected ER ligands **9** and **10** and the carboxylic acid functional group of the combretastatin acrylic acid analogues **13**–**24** can undergo coupling reactions using DCC and HOBt, forming an amide linkage to afford silyl-ether protected conjugated compounds in high yields (See Scheme 3). The silyl-ether protecting groups were then removed using TBAF to afford the phenolic conjugates **27**–**46** as ~1:1 (*E/Z*) isomeric mixtures in high yield (88%–94%) following chromatographic purification. In the ^1^H NMR spectrum the characteristic protons of the ethyl group are observed in the region 0.90–0.96 ppm (CH_3_) and 2.40–2.50 ppm (CH_2_), the amine methyl group is found between 3.04 and 3.25 ppm, while the methylene protons of the basic side chain are identified at 3.53–4.20 ppm. The amide compounds **47**–**53** were also prepared by reaction of the acrylic acids **13**, **14**, **21** and **23** with pyrrolidine and piperidine using the Mukaiyama reagent (2-chloro-1-methylpyridinium iodide) as the coupling reagent. The amines **54** and **55** were obtained by reduction of the corresponding nitro compounds **50** and **51** using iron in hydrochloric acid.

### 3.2. Biochemical Studies

#### 3.2.1. Antiproliferation and Cytotoxicity Studies

The ability of the compounds **27**–**46** synthesised to inhibit the proliferation of the human breast tumour MCF-7 cell line was investigated using a standard MTT cell viability assay while the compounds were concurrently tested to assess the extent of their cytotoxicity using a LDH assay [66]. The MCF-7 is an ER-positive human cancer cell line; where ER is overexpressed in the cell line [67]. Selected conjugates were also evaluated using ER-negative MDA human cancer cell-line in order to assess any possible ER-selectivity of the conjugates. The IC_50_ value obtained for the control **26** (CA4) in this assay is 0.008 μM for MCF-7 and is in good agreement with the reported values for CA4 using the MTT assay on human MCF-7 breast cancer cell lines [59,68,69]; the IC_50_ value obtained for the control endoxifen **11** in this assay is 0.029 nM while the reported IC_50_ value for endoxifen in the MCF-7 cell line is 50 nM [70].

In general, combretastatin acrylic acid derivatives have a lower antiproliferative potency compared to the lead compound, combretastatin A-4 (CA4), **26** (IC_50_ = 8 nM), (see Table 1). The acrylic acid **13** displayed the highest antiproliferative action in the series, with IC_50_ value of 0.120 μM while all the analogues tested showed negligible cytotoxic affects in the LDH assay, (0% cell death at 10 μM). The substitution pattern in the A and B rings of **13** are similar to that of the potent combretastatin CA4. It is interesting that the compound **23**, in which the A ring having the 3,4,5-trimethoxyphenyl substituent is positioned on the carbon β to the acrylic acid is inactive with IC_50_ > 50 μM. The acrylic acids **14**, **16**, **21** and the ester **24** all demonstrated IC_50_ values less than 10 μM against MCF-7 cell line. The antiproliferative activity of acrylic acids has been previously reported [23]. The acrylic acid secondary amides **47**–**55** prepared from pyrrolidine and piperidine were also evaluated for antiproliferative activity in the MCF-7 cell line (Table 1). The compounds were found to be of low potency, e.g., the amide **49** (IC_50_ = >50 μM in comparison with the acrylic acid **13** (IC_50_ = 0.120 μM) which suggested that a more bulky amide such as endoxifen may be required for activity in MCF-7 cells.

The conjugates 27–46 displayed a wide range of antiproliferative activity with IC_50_ values in the range 0.005–6.15 µM (see Table 2). In all examples, the coupling of the acrylic acid to the endoxifen 11 or the novel hydroxyendoxifen 12 resulted in an increase in the antiproliferative activity when compared to the corresponding acrylic acid and amides 47–55. The most potent conjugate 28 displayed antiproliferative action in the MCF-7 cell-line with an IC_50_ value of 5 nM—a value greater than either of the values for combretastatin A-4 (IC_50_ = 8 nM), endoxifen 11 (29 nM) and the novel hydroxyendoxifen analogue 12 (IC_50_ = 28 nM). In many examples, coupling of the acrylic acid to the novel hydroxyendoxifen 12 resulted in a more potent conjugate product than the coupling of the same acid with endoxifen 11, (e.g., IC_50_ of compound 27 = 0.033 nM, IC_50_ of compound 28 = 0.005 nM). Since there is much interest in combretastatin analogues having the 3-amino-4-methoxy substituion patern in Ring B [52] it was of interest in this work to evaluate the conjugates 45 and 46. While moderate activity was observed, (IC_50_ = 0.959 and 6.15 for compounds 45 and 46 respectively, these compounds were not as potent as the corresponding Ring B 3-hydroxy-4-methoxy substitute compounds 27 and 28. The ring B 3-fluoro-4-methoxy substituted compounds 43 and 44 displayed impressive antiproliferative activity with IC_50_ values of 180 and 194 nM respectively.

Conjugate compounds **29** and **30** substituted with 4-methoxy group in Ring B of the acrylic acid component, also display potent activity with IC_50_ values of 87 nM and 43 nM respectively. Compound **31** which contains 3,4,5-trimethoxy substitution pattern in the Ring B acrylic acid component and the 4-methoxy substituent on Ring A demonstrate a reduced activity (IC_50_ = 2.08 µM), however in compound **32** which is the corresponding conjugate with hydroxyendoxifen, the antiprolifertive activity is determined at 41 nM, indicating the potent effect on activity of conjugation with hydroxyendoxifen. Inclusion of the 4-thiomethyl substituent or the 3,4-methylenedioxy substitution on Ring B of the acrylic acid component resulted in potent activity for the hydroxyendoxifen conjugated compounds **34** and **40** (IC_50_ = 49 nM and 64 nM respectively). Introduction of the 2-naphthyl Ring B as the acrylic acid component resulted in a reduced antiproliferative effect for compounds **36** and **37** (IC_50_ values = 1.51 and 3.62 nM respectively). It would have been interesting to determine whether the addition of estradiol would alter the IC_50_ values in Table 2 and thus definitively determine whether the antiproliferative effects observed had a ER-mediated component or whether these effects were solely mediated through the combretastatin component of the conjugate.

The most active compounds from the MCF-7 assay were selected for evaluation in the ER-negative MDA-MB-231 human breast cancer cell line. The selected conjugates (**27**–**36**, **41**–**46**) as expcted displayed reduced antiproliferative activity (micromolar IC_50_ values) compared with results obtained for the ER-positive MCF-7 cell line, indicating that the conjugates show a selectivity for the ER expressing cell line (see Table 3). The most active compound was identified as conjugate **34** with IC_50_ value of 0.68 µM, while compounds compounds **27**, **28**, **35** and **44** displayed moderate activity with IC_50_ values of 2.67, 2.48, 2.16 and 1.85 µM, respectively, all of which are more potent than tamoxifen, (IC_50_ = 20 µM) and 4-hydroxytamoxifen (IC_50_ = 18 µM) in this ER negative cell line.

#### 3.2.2. Estrogen Receptor Binding

The receptor binding affinity for the lead conjugate **28** was investigated in both ERα and ERβ using a fluorescence polarisation based competitive binding assay. Competitive binding affinity experiments were carried out using purified baculovirus-expressed human ERα and ERβ and fluoromone, a fluorescein labelled estrogen ligand [33,34]. The endogenous ligand β-estradiol was used as the positive control in the experiments. The lead conjugate displayed potent binding in both ER isoforms (IC_50_ value for ERα: 0.9 nM; IC_50_ value for ERβ: 4.7 nM). These binding values are greater than the endogenous ligand estradiol, 4-hydroxytamoxifen, endoxifen **11** and significantly greater than that of the parent ER-ligand, hydroxyendoxifen **12** (ERα: IC_50_ 44.1 nM; ERβ: IC_50_ 39.7 nM) (see Table 4).

#### 3.2.3. NCI 60 Cell Line Screen

On the basis of potency, compound **28** was evaluated in the National Cancer Institute (NCI, Bethesda, MD, USA) Division of Cancer Treatment and Diagnosis(DCTD)/Developmental Therapeutics Programme(DTP) in which the activity of the compound is determined using a 60-cell line screen facility of different cancer cell lines of diverse tumour origin [71]. Compound was tested for inhibition of growth(GI_50_) and cytotoxicity(LC_50_). These studies were performed at the NCI as part of their drug screening programme. Initially, the compound was evaluated against the 60 cell lines at a single dose of 10 μM; if significant growth inhibition was exhibited the compound was evaluated against the 60 cell panel at a further five concentration levels, 0.01–100 μM, (see Table 5). In the one-dose screen, **28** displayed low lethality and a mean growth inhibition value of 98.73% over the 60 cell lines. **28** displayed very high growth inhibition in the cell lines of non-small cell lung cancer NCI-H23 (97%) and NCI-H460 (98%); colon cancer HCT-116 (98%); breast cancer MCF-7 (94%) and MDA-MB-435 (91%); ovarian cancer OVCAR-8 (97%) and SK-OV-3 (95%); leukaemia RPMI-8226 (91%); renal cancers ACHN (96%), CAKI-1 (91%), RXF 393 (95%) and SN12C (90%); melanoma SK-MEL-2 (97%) and CNS cancer U251 (93%). The compound caused between 80%–89% growth inhibition in a further 14 cell lines and was very toxic to the non-small cell lung cancer NCI-H226 (73% lethality) and melanoma SK-MEL-5 (60% lethality) cell lines.

In the five-dose screen, **28** displayed low micromolar GI_50_ (IC_50_) values for the majority of the 60 cancer cell lines. However, **28** demonstrated a high selectivity towards MCF-7 breast cancer with a GI_50_ (IC_50_) value of 9.5 nM and a LC_50_ value greater than 50 μM for this cell line. The mean GI_50_ value compound **28** across all 60 panel cell lines is 1.45 µM, (log GI_50_ = −5.79). As **28** has been shown to be a high-affinity ER-binding ligand, the high specificity towards MCF-7 cells is most probably due to its selective antagonistic effects on the over-expressed ER within this cell-line. This is an impressive and promising result as it confirms the suitability of the selected prototypes and project strategy for the possible therapeutic application of the ER-conjugates synthesised in the study. The LC_50_ value is a measure of the cytotoxicity of the compound in each cell line; the mean LC_50_ for compound **28** is 46.77 µM and was greater than 50 µM in all but 4 cell lines. (NCI-H23: 48.9 µM; COLO 205: 26.2 µM; SK-MEL-5: 34.5 µM; A498: 32.6 µM). The mean LC_50_ for 28 across all 60 panel cell lines is 46.77 µM; (see Table 5).

The NCI provide the pattern recognition algorithm COMPARE [72,73]. The unique complexity of a 60-cell line dose response produced by a given compound results in a biological response pattern which can be utilized in pattern recognition algorithms. Using the COMPARE algorithms, it is possible to assign a putative mechanism of action for the screened compound, or to determine that the response pattern is unique and not similar to that of any of the standard prototype compounds included in the NCI database. In addition, following characterization of various cellular molecular targets in the 60-cell lines, it may be possible to select compounds most likely to interact with a specific molecular target. Generally, a correlation coefficient greater than 0.6 is considered a positive correlation [74]. For the five-dose screen COMPARE analyses, the highest correlation coefficients achieved for compound **28**, from both the common anticancer agent database and the comprehensive database, were 0.934 in relation to the potent antiviral and anticancer agent, Didemnin B [75] which selectively induces apoptosis through dual inhibition of PPT1 and EEF1A1, 0.879 for the antileukemic agent and protein synthesis inhibitor, Bruceantin [76] and 0.430 in relation to the antimitotic/anti-tumour agent Rhizoxin **47** [77,78]. Correlation values are Pearson correlation coefficients based on GI_50_ mean graphs. These correlation values demonstrate a high similarity of activity and suggest a common mechanism of action between the agents. The antiproliferative activity observed for the conjugate compound **28** indicated that there is a significant therapeutic window between the concentration required for cancer cell growth inhibition and the concentration that is toxic to MCF-7 cells.

#### 3.2.4. Ishikawa Cell Line Study

**Figure 2:**
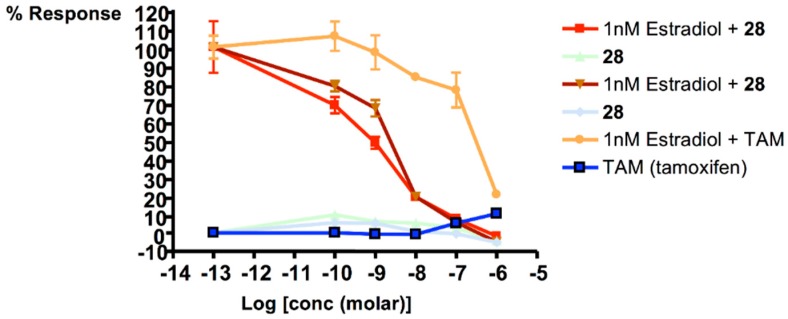
Effect on alkaline phosphatase activity of conjugate **28** and tamoxifen in Ishikawa cells.

The Ishikawa assay is used to measure estrogen stimulation of alkaline phosphatase enzyme activity (AlkP) by the Ishikawa cell line of human endometrial adenocarcinoma cells [35]. The Ishikawa assay provides a measure of the agonist activity of a compound and was carried out on the lead conjugate, 28. Tamoxifen was used as a reference compound in the assay. The effect of the compounds on estrogen stimulation within the Ishikawa cells can be seen in Figure 2, which is representative of an experiment that was carried out three times. Tamoxifen displays some estrogenic activity at higher concentrations. When Tamoxifen is dosed with a 1 nM estradiol spike, there is estrogen stimulation at low concentration while a reduction occurs as the concentration of Tamoxifen is increased. The conjugate was dosed in duplicate. The conjugate 28 is similar to Tamoxifen when dosed individually. However, the conjugate appears not to display any estrogenic activity at higher concentrations, which is desirable. This may suggest that the conjugate is acting as a full antagonist. When 28 was dosed as a mixture with estradiol, there is a reduction in estrogenic stimulation at low concentrations and a more pronounced reduction in estrogen stimulation over the range of concentrations when compared with the Tamoxifen mixture. This preliminary result is promising as it demonstrates that the conjugate 28 does not display estrogenic stimulation in the cell line and shows reduced estrogenic stimulation compared to Tamoxifen over the concentration range investigated.

#### 3.2.5. Stability Studies

The stability of the selected target compounds **27**, **31** and **32** was evaluated in phosphate buffer at pH values in the range 4–9 and the half-life was determined to be greater than 24 h for each compound at these pH values. The compounds were found to stable without any significant degradation of the conjugates suggesting that the combretastatin fragment remains intact at physiological pH; and indicating that the combretastatin component of the conjugate has a role in displacing helix-12 of the ER resulting in the potent (and possibly pure) antagonistic activity.

### 3.3. Molecular Modelling

The intricate molecular basis of estrogen receptor agonism and antagonism has been well studied [1,79,80,81]. Phenol groups, a common structural motif to many ER ligands, take part in direct hydrogen bonding with the same key residues in the ER (i.e., Glu353, Arg 394 and His524 in human ERα). Within the ER ligand-binding domain (LBD), ligand recognition is achieved through a combination of specific hydrogen bonds and the complementarity values of the binding cavity with the relevant ligands non-polar character. Antagonistic ligands such as Tamoxifen and 4-hydroxytamoxifen contain a basic side chain group too large to be accommodated in the binding cavity, resulting in the displacement of the Helix-12 in the ER protein structure. In particular, the positioning of Helix-12 in the ER is key to the recruitment of coactivators/corepressors and determining the overall nature of the ligand effect [81].

The interactions of compound **28** was modelled in both ERα and ERβ isoforms in order to rationalise the biochemical data obtained for this compound. The modelling strategy protocol involved calculating the appropriate protonation state at physiological pH using MARVIN [82]; the determination of the lowest energy conformer using MACROMODEL [38]; the generation of an ensemble of low-energy conformers using OMEGA [39], followed by the docking and scoring of these conformers using FRED [40]. Graphical manipulations were carried out using DS VISUALIZER [83]. High-ranking docking solutions were investigated—noting favourable interactions between the conjugate and the residues in the ER isoform ligand binding domains.

**Figure 3:**
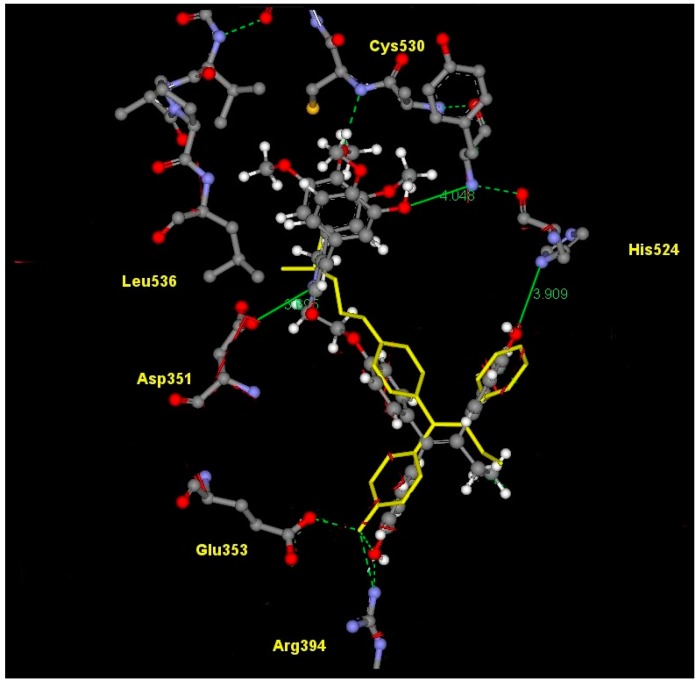
Predicted binding mode of compound 28 in the ligand binding domain of ERα. Highest-ranking docking orientation and predicted receptor interactions for **28** (ball-and-stick representation) in ERα compared to the crystal solution for 4-hydroxytamoxifen (yellow); pdb 3ERT [41]. (H-bond interactions are illustrated as broken lines).

The top-ranking docking solution in human ERα for compound **28** is shown in Figure 3. For comparison, the 4-hydroxytamoxifen bound ligand (reference pdb: 3ERT) [41] is displayed in yellow while the docked confirmations are coloured by element type (carbon = grey, hydrogen = white, oxygen = red, nitrogen = blue). In Figure 3, the Glu353 and Arg394 residues form hydrogen bond interactions with the phenol group, similar to that of 4-hydroxytamoxifen. In this docked solution the additional phenol group of **28** is found 3.9 Å away from the His524 residue and does not form a strong H-bond. The Asp351 residue is located 3.7 Å away from the amide nitrogen of **28**, which would translate to a weak interaction. The Cys530 residue forms a H-bond with the C-4 methoxy group on the A-ring of the combretastatin fragment as illustrated in Figure 3. The increased bulk of the ligand side chain of the conjugate **28** may displace Helix-12 to a significant extent and could explain the antagonistic activity and impressive binding affinity of this lead compound **28** (0.9 nM in ERα; 4.7 nM in ERβ).

**Figure 4:**
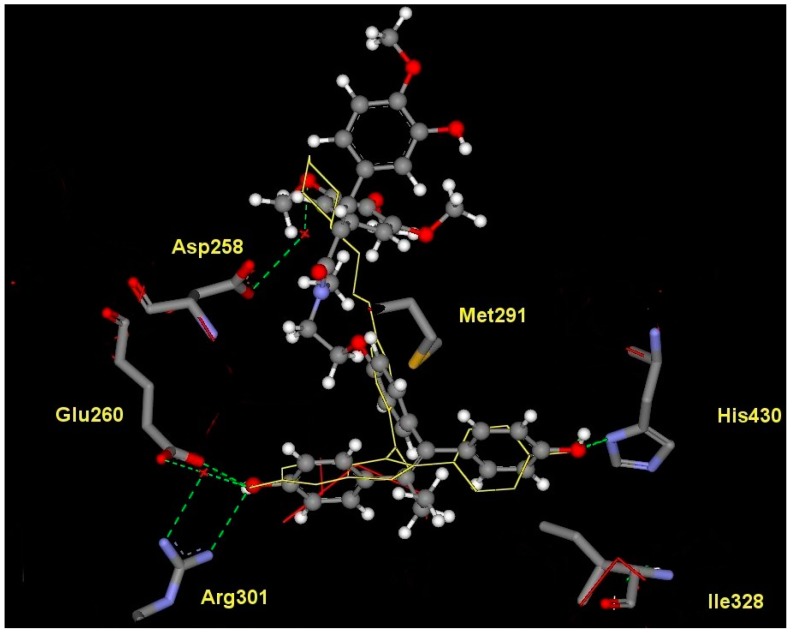
Predicted binding mode of compound **28** in the ligand binding domain of ERβ. Highest-ranking docking orientation and predicted receptor interactions for **28** (ball-and-stick representation) in ERβ compared to the crystal solution for 4-hydroxytamoxifen (yellow) (pdb 1QKN) [42]. (H-bond interactions are illustrated as broken lines).

The top-ranking docking solutions in rat ERβ for compound **28** is shown in Figure 4. For comparison, the raloxifene bound ligand (reference pdb: 1QKN) [42] is displayed in yellow while the docked confirmations are coloured by element type (carbon = grey, hydrogen = white, oxygen = red, nitrogen = blue). In Figure 4, the residues Glu260 and Arg301 form favourable hydrogen bond interactions with the phenol group, similar to that of 4-hydroxytamoxifen. The additional group present on **28** forms an additional hydrogen bond interaction with the His430 residue. There is good agreement/overlap of the raloxifene core and the endoxifen moiety of **28**. A methoxy group present on the combretastatin moiety of **28** forms another favourable hydrogen bond through a water molecule with the Asp258 residue within the LBD. This interaction could help “lock in” the ligand within the cavity site, resulting in the good binding affinity. Additional waters unaccounted for in this determined crystal structure may introduce further favourable hydrogen bond interactions with the other methoxy groups of the combretastatin moiety through a network of water molecules. The alkyl groups of Ile328 are in close proximity to a phenyl group present on the endoxifen moiety, which could produce a lipophilic-lipophilic interaction. The bulky side chain of **28** would have a greater degree of rigidity due to the presence of the amide linkage and may displace Helix-12 significantly in ERβ to produce antiproliferative effects observed.

## 4. Conclusions

A series of prototype ER-ligand conjugates **27**–**46** were successfully synthesised incorporating an endoxifen-combretastatin hybrid scaffold with potential SERM properties. A number of these novel compounds displayed potent antiproliferation activity in the MCF-t human breast cancer cell line with low cytotoxicity values. The conjugate **28** in the series was the most promising compound in the study, with potent antiproliferative activity in MCF-7 human breast cancer cell line (IC_50_: 5 nM), low cytotoxicity and impressive ER competitive binding IC_50_ values (ERα: IC_50_ 0.9 nM; ERβ: IC_50_ 4.7 nM). A number of these conjugate compounds also display activity in the ER negative cell line MDA-MB-321 at low micromolar and sub-micromolar concentrations, e.g., compound **34** (IC_50_ = 0.68 µM). Preliminary stability studies show that these conjugates do not degrade significantly at physiological pH values. Interestingly, from a structural biology standpoint, this result indicates that the ER can tolerate a large substituent such as the combretastatin analogue, at the basic side chain of the triarylethylene ER ligand scaffold without detrimental effect on the overall antiproliferative and ER binding characteristics and biochemical activity. The lead conjugate compounds **27** and **28** are currently under further investigation by our research group to further explore its mechanism of action and potential applications in the development of useful ER ligands. These compounds demonstrate a novel and interesting class of ER ligand and may have potential future application as medicinal agents for anticancer therapy.

## Figures and Tables

**Table 1:** Antiproliferative effects of acrylic acids **13**–**25** in MCF-7 human breast cancer cells ^a^.

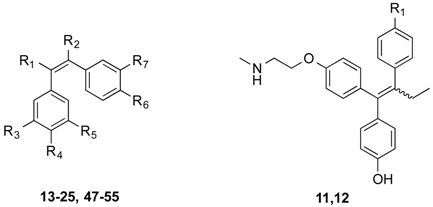								
Compound	R_1_	R_2_	R_3_	R_4_	R_5_	R_6_	R_7_	IC_50_ (μM) ^b^
**13**	COOH	H	OMe	OMe	OMe	OMe	OH	0.120
**14**	COOH	H	OMe	OMe	OMe	OMe	H	7.80
**15**	COOH	H	OMe	OMe	OMe	OMe	F	55.1
**16**	COOH	H	OMe	OMe	OMe	OMe	NH_2_	6.70
**17**	COOH	H	OMe	OMe	OMe	Br	H	>50
**18**	COOH	H	OMe	OMe	OMe	F	H	>50
**19**	COOH	H	OMe	OMe	OMe	Me	H	26.1
**20**	COOH	H	OMe	OMe	OMe	SMe	H	10.6
**21**	COOH	H	OMe	OMe	OMe	OMe	NO_2_	2.50
**22**	COOH	H	OMe	OMe	OMe	-C_4_H_4_-		15.3
**23**	H	COOH	OMe	OMe	OMe	OMe	H	>50
**24**	CO_2_Me	H	OMe	OMe	OMe	OMe	OH	2.80
**25**	H	COOH	-OCH_2_O-		H	OMe	H	>50
**47**	H	CO-Pi	OMe	OMe	OMe	OMe	H	> 50
**48**	H	CO-Py	OMe	OMe	OMe	OMe	H	>50
**49**	CO-Pi ^e^	H	OMe	OMe	OMe	OMe	OH	>50
**52**	CO-Py ^f^	H	OMe	OMe	OMe	OMe	H	>50
**53**	CO-Pi ^e^	H	OMe	OMe	OMe	OMe	H	^g^
**54**	CO-Py ^f^	H	OMe	OMe	OMe	OMe	NH_2_	>50
**55**	CO-Pi ^e^	H	OMe	OMe	OMe	OMe	NH_2_	^h^
**26** (**CA4**) ^c^	H	H	OMe	OMe	OMe	OMe	OH	0.008
**11** ^d^	H	-	-	-	-	-	-	0.029
**12**	OH	-	-	-	-	-	-	0.028

^a^ Mean IC_50_ values of compounds for their antiproliferative effects and percent cytotoxicity on a human MCF-7 breast cancer cell line. ^b^ IC_50_ values are half maximal inhibitory concentrations required to block the growth stimulation of MCF-7 cells. Values are an average of at least three experiments performed in triplicate with typical standard errors below 15%. ^c^ The IC_50_ value obtained for **26** (CA4) in this assay is 8 nM for MCF-7 and is in good agreement with the reported values for CA4 using the MTT assay on human MCF-7 breast cancer cell lines(see refs. [59,68,69]). ^d^ The reported IC_50_ value for endoxifen in the MCF-7 cell line is 50 nM [70]. ^e^ Pi piperidine, ^f^ Py = pyrrole. ^g^ 35% growth inhibition for compound **53** at 10 μM. ^h^ 34% growth inhibition for compound **55** at 10 μM.

**Table 2:** Antiproliferative effects of conjugate compounds **27**–**46** in MCF-7 human breast cancer cells ^a^.

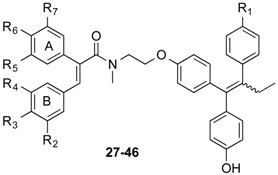									
Compound	R_1_	R_2_	R_3_	R_4_	R_5_	R_6_	R_7_	IC_50_ (μM) ^b^	% Cell Death at 10 μM ^c^
**27**	H	OH	OMe	H	OMe	OMe	OMe	0.033	10
**28**	OH	OH	OMe	H	OMe	OMe	OMe	0.005	17
**29**	H	H	OMe	H	OMe	OMe	OMe	0.087	12
**30**	OH	H	OMe	H	OMe	OMe	OMe	0.043	19
**31**	H	OMe	OMe	OMe	H	OMe	H	2.08	1
**32**	OH	OMe	OMe	OMe	H	OMe	H	0.041	16
**33**	H	-OCH_2_O-		H	H	OMe	H	1.47	8
**34**	OH	-OCH_2_O-		H	H	OMe	H	0.049	6
**35**	H	-C_4_H_4_-		H	OMe	OMe	OMe	1.51	0
**36**	OH	-C_4_H_4_-		H	OMe	OMe	OMe	3.62	3
**37**	H	H	Me	H	OMe	OMe	OMe	1.75	2
**38**	OH	H	Me	H	OMe	OMe	OMe	0.372	12
**39**	H	H	SMe	H	OMe	OMe	OMe	0.790	3
**40**	OH	H	SMe	H	OMe	OMe	OMe	0.064	2
**41**	H	H	Br	H	OMe	OMe	OMe	1.03	5
**42**	OH	H	Br	H	OMe	OMe	OMe	2.35	7
**43**	H	F	OMe	H	OMe	OMe	OMe	0.180	2
**44**	OH	F	OMe	H	OMe	OMe	OMe	0.194	10
**45**	H	NH_2_	OMe	H	OMe	OMe	OMe	0.959	8
**46**	OH	NH_2_	OMe	H	OMe	OMe	OMe	6.15	6
**26 (CA4)** ^d^	-	-	-	-	-	-	-	0.008	13
**11** ^e^	-	-	-	-	-	-	-	0.029	23
**12**	-	-	-	-	-	-	-	0.028	11

^a^ Mean IC_50_ values of compounds for their antiproliferative effects and percent cytotoxicity on a human MCF-7 breast cancer cell line using the MTT assay. ^b^ IC_50_ values are half maximal inhibitory concentrations required to block the growth stimulation of MCF-7 cells. Values are an average of at least three experiments performed in triplicate with typical standard errors below 15%. ^c^ Lactate Dehydrogenase assay: Following treatment of the cells, the amount of LDH was determined using LDH assay kit from Promega. Data is presented as % cell lysis at compound concentration of 10 µM [66]. ^d^ The IC_50_ value obtained for **26** (CA4) in this assay is 8 nM for MCF-7 and is in good agreement with the reported values for CA4 using the MTT assay on human MCF-7 breast cancer cell lines ([59,68,69]). ^e^ The reported IC_50_ value for endoxifen **11** in the MCF-7 cell line is 50 nM [70].

**Table 3:** Antiproliferative effects of selected conjugate compounds in MDA-MB-231 human breast cancer cells ^a^.

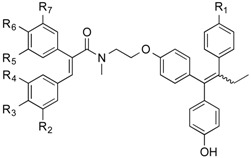									
Compound	R_1_	R_2_	R_3_	R_4_	R_5_	R_6_	R_7_	IC_50_ (μM) ^b^	% Cell Death at 10 μM ^c^
**27**	H	OH	OMe	H	OMe	OMe	OMe	2.67	17
**28**	OH	OH	OMe	H	OMe	OMe	OMe	2.48	15
**29**	H	H	OMe	H	OMe	OMe	OMe	30.6	2
**30**	OH	H	OMe	H	OMe	OMe	OMe	6.29	7
**31**	H	OMe	OMe	OMe	H	OMe	H	51.0	0
**32**	OH	OMe	OMe	OMe	H	OMe	H	4.09	0
**33**	H	OCH_2_O		H	H	OMe	H	5.23	0
**34**	OH	OCH_2_O		H	H	OMe	H	0.68	16
**35**	H	-C_4_H_4_-		H	OMe	OMe	OMe	2.16	0
**36**	OH	-C_4_H_4_-		H	OMe	OMe	OMe	4.32	0
**41**	H	H	Br	H	OMe	OMe	OMe	>50	0
**42**	OH	H	Br	H	OMe	OMe	OMe	>50	9
**43**	H	F	OMe	H	OMe	OMe	OMe	30.3	0
**44**	OH	F	OMe	H	OMe	OMe	OMe	1.85	14
**45**	H	NH_2_	OMe	H	OMe	OMe	OMe	7.11	0
**46**	OH	NH_2_	OMe	H	OMe	OMe	OMe	49.7	0
**Tamoxifen**								20	0
**Hydroxytamoxifen**								18	0
**26 (CA4)**								0.043	4

^a^ Mean IC_50_ values of compounds for their antiproliferative effects and percent cytotoxicity on a human MDA breast cancer cell line. ^b^ IC_50_ values are half maximal inhibitory concentrations required to block the growth stimulation of MDA-MB-231 cells. Values are an average of at least three experiments performed in triplicate with typical standard errors below 15%. **^c^** Lactate Dehydrogenase assay: Following treatment of the cells, the amount of LDH was determined using LDH assay kit from Promega. Data is presented as % cell lysis at compound concentration of 10 μM [66].

**Table 4:** Estrogen receptor binding affinities for compounds **11**, **12** and **28** ^a^.

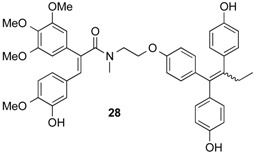			
Compound	IC_50_ (nM) ^a,b^		Selectivity (α/β)
	ERα	ERβ	
**28**	1	5	5.22
**11** (Endoxifen)	47	118	2.52
**12** (Hydroxyendoxifen)	44	40	0.90
4-Hydroxytamoxifen	30	26	0.88
Estradiol	6	6	0.98

^a^ Mean IC_50_ values of compounds for their competitive binding in ERα and ERβ isoforms. ^b^ Values are an average of at least nine replicate experiments, for ERα with typical standard errors below 15%, and six replicate experiments for ERβ, with typical standard errors below 15%.

**Table 5:** Results of the in vitro human cancer cell growth inhibition for compound **28** ^a^. Antiproliferative evaluation of compound **28** in the NCI60 cell line in vitro screen.

Cell Line	Compound 28	Compound 28	CELL LINE	Compound 28	Compound 28
	GI% (10^−5^ M) ^a^	GI_50_ (μM) ^b^		GI% (10^−5^ M) ^a^	GI_50_ (μM) ^b^
*Leukemia*			*Melanoma*		
CCRF-CEM	72.48	1.77	LOX IMV1	1.55	87.57
HL-60 (TB)	100 ^d,f^	1.4	MALME-3M	1.76	76.76
K-562	86.1	2.57	M14	1.68	100 ^d,p^
MOLT-4	64.31	1.52	MDA-MB-235	0.747	90.98
RPMI-8226	90.65	0.896	SK-MEL-2	8.67	97.42
SR	69.03	2.35	SK-MEL-28	1.65	76.19
*Non-Small Cell Lung Cancer*			SK-MEL-5	0.837	100 ^d,q^
A549/ATCC	86.97	2.88	UACC-257	2.83	100 ^d,r^
EKVX	85.11	2.01	UACC-62	0.922	100 ^d,s^
HOP-62	81.99	2.86	*Ovarian Cancer*		
HOP-92	86.19	0.764	IGROV1	2.3	100 ^d,t^
			OVCAR-3	1	100 ^d,u^
NCI-H226	100 ^d,g^	1.18	OVCAR-4	2.59	81.47
NCI-H23	96.76	2.19	OVCAR-5	3.21	82.75
NCI-H332M	81.7	2.02	OVCAR-8	2.02	97.21
NCI-H460	98.38	1.42	NCI/ADR-RES	6.02	21.31
NCI-H552	100 ^d,h^	1.39	SK-OV-3	1.66	95.08
*Colon Cancer*			*Renal Cancer*		
COLO 205	100 ^d,i^	2.15	786-0	1.91	100 ^d,v^
HCT-2998	100 ^d,j^	1.25	A498	2.94	80.37
HCT-116	97.57	1.8	ACHN	1.92	96.43
HCT-15	100 ^d,k^	1.53	CAKI-1	1	91.46
HT29	100 ^d,l^	1.16	RXF 393	1.89	95.02
KM12	100 ^d,m^	1.26	SN12C	1.8	90.3
SW-620	81.32	1.21	TK-10	1.65	100 ^d,w^
*CNS Cancer*			UO-31	2.66	89.77
SF-268	84.22	1.69	*Breast Cancer*		
SF295	73.4	1.74	MCF-7	0.00998	94.3
SF539	100 ^d,n^	1.18	MDA-MB-231/ATCC	1.61	73.55
SNB-19	81.49	1.72	HS 578T	2.04	64.8
SNB-75	60.65	5.96	BT-549	1.12	100 ^d,x^
U251	93.37	1.74	T-47D	Nd ^c^	100 ^d,y^
*Prostate cancer*			MDA-MB-468	1.12	100 ^d,z^
PC-3	86.32	1.73			
DU-145	100 ^d,o^	1.66			
MG-MID (µM) ^e^		1.45			

^a^ Data obtained from NCI in vitro human tumour cell screen at 10 µM concentration. ^b^ GI_50_ is the molar concentration of the compound causing 50% inhibition of growth of the tumour cells; **^c^** Nd: Not determined; ^d^ Cytotoxic effect; ^e^ MG-MID: the mean of GI_50_ values over all cell lines for the tested compound. ^f^ Cell Growth Percent −13.32%. ^g^ Cell Growth Percent −72.63%. ^h^ Cell Growth Percent −22.04%. ^i^ Cell Growth Percent −41.64%. ^j^ Cell Growth Percent −16.22%. ^k^ Cell Growth Percent −15.41%. ^l^ Cell Growth Percent −32.91%. ^m^ Cell Growth Percent −51.93%. ^n^Cell Growth Percent −6.56%. ^o^ Cell Growth Percent −19.73%. ^p^ Cell Growth Percent −20.28%. ^q^ Cell Growth Percent −59.57%. ^r^ Cell Growth Percent −0.60%. ^s^ Cell Growth Percent −19.77%. ^t^ Cell Growth Percent 35.74%. ^u^ Cell Growth Percent 30.95%. ^v^ Cell Growth Percent −26.91%. ^w^ Cell Growth Percent −29.83%. ^x^ Cell Growth Percent −23.96%. ^y^ Cell Growth Percent 9.09%. ^z^ Cell Growth Percent −34.28%.
